# Balancing speed and accuracy of polyclonal T cell activation: a role for extracellular feedback

**DOI:** 10.1186/1752-0509-6-111

**Published:** 2012-08-27

**Authors:** Yonatan Savir, Nir Waysbort, Yaron E Antebi, Tsvi Tlusty, Nir Friedman

**Affiliations:** 1 The Simons Center for Systems Biology, Institute for Advanced Studies, Princeton, New Jersey, 08540, USA; 2 Department of Immunology, Weizmann Institute of Science, Rehovot, 76100, Israel; 3 Department of Systems Biology,, Harvard Medical School Harvard Medical School, Warren Alpert Building, Room 555B 200 Longwood Avenue, Boston, MA, 02115, USA; 4 Division of Biology, Caltech, Pasadena, CA, 91125, USA

**Keywords:** Systems immunology, Positive feedback, Speed vs. accuracy, IL-2, CD25

## Abstract

**Background:**

Extracellular feedback is an abundant module of intercellular communication networks, yet a detailed understanding of its role is still lacking. Here, we study interactions between polyclonal activated T cells that are mediated by IL-2 extracellular feedback as a model system.

**Results:**

Using mathematical modeling we show that extracellular feedback can give rise to opposite outcomes: competition or cooperation between interacting T cells, depending on their relative levels of activation. Furthermore, the outcome of the interaction also depends on the relative timing of activation of the cells. A critical time window exists after which a cell that has been more strongly activated nevertheless cannot exclude an inferior competitor.

**Conclusions:**

In a number of experimental studies of polyclonal T-cell systems, outcomes ranging from cooperation to competition as well as time dependent competition were observed. Our model suggests that extracellular feedback can contribute to these observed behaviors as it translates quantitative differences in T cells’ activation strength and in their relative activation time into qualitatively different outcomes. We propose extracellular feedback as a general mechanism that can balance speed and accuracy – choosing the most suitable responders out of a polyclonal population under the clock of an escalating threat.

## Background

**Figure 1:**
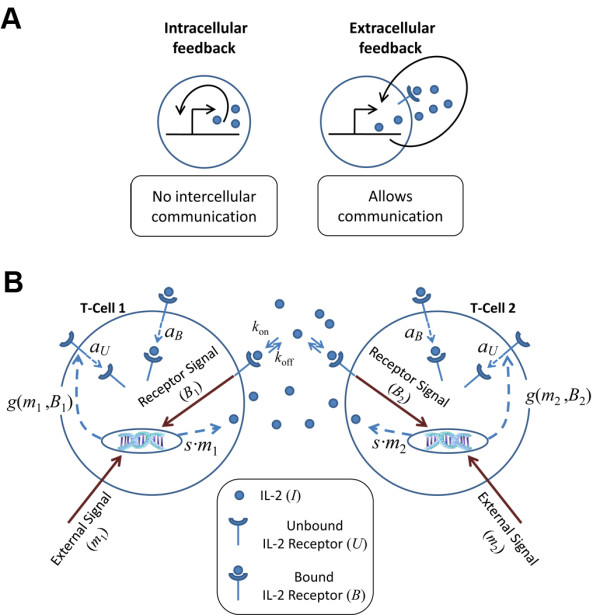
**The extracellular feedback module.** (**A**) Extracellular feedback allows for communication between cells. Intracellular feedback (left) relies on sensing a molecule’s level inside the cell. Extracellular feedback (right) relies on sensing the level of a molecule that is secreted to the environment, thus allowing neighboring cells to sense it and engage in the feedback themselves. Sensing the cell’s environment requires receptors, and thus the feedback can modulate the rate of production of the signaling molecule and/or its receptor. (**B**) Schematics of the IL-2 extracellular feedback system in T-cell activation. T-cells are activated through binding of the T-cell receptors (TCRs) by their cognate peptide-MHC complex. TCR binding induces signals with different strengths, depending on binding affinity and number of bound receptors. Activation signal strength is denoted by *m*_1_ and *m*_2_ for the two interacting cells. Following TCR signal, the cytokine IL-2 is secreted with a rate that is proportional to the TCR signal level (*s·m*). In the absence of IL-2, the unbound IL-2 receptor (IL-2R) is generated at a low constitutive rate and is internalized at a rate *a*_*U*_, thus is present in low numbers (~100 molecules per cell). The binding of IL-2 to its receptor causes up-regulation of IL-2R synthesis. The overall IL-2R production rate, *g*(*m*, *B*), depends on the TCR signal strength, *m*, and on the number of bound IL-2R, *B*. The IL-2R internalization rate is also increased upon binding of IL-2 (*a*_*B*_). The IL-2 molecule is released to the intercellular space and can be captured by any T-cell. Thus, two processes are in effect: autocrine and paracrine positive feedbacks. These processes allow two nearby T-cells, potentially with different TCR specificities, to communicate and influence each other.

Positive feedback is common in biological and ecological systems [1,2], and has been shown to generate various behaviors including bistability [3] and hysteresis [4]. *Intracellular* positive feedback, which serves as a common module of gene regulatory networks, has been extensively studied both theoretically and experimentally [5-8]. However, in multicellular systems, positive feedback can be mediated by a secreted molecule which acts either in an autocrine fashion (on the secreting cell) or paracrinally (on nearby cells) [9]. This leads to a collective cellular response, during which cells communicate their state to nearby cells using the extracellular signaling molecule. Although *extracellular* positive feedback serves as a basic building block of intercellular communication networks (Figure 1A), a detailed understanding of its function is still missing.

The immune system offers numerous examples of extracellular positive feedback mediated by cytokines (small secreted proteins). A specific example is provided by the cytokine Interlukin-2 (IL-2) through its roles in the process of T cell activation. T cells are activated in response to binding of an antigen to their T cell receptor (TCR). Upon TCR engagement and receipt of appropriate co-stimulatory signals T cells secrete IL-2, which serves as a major T cell growth factor that promotes their commitment to proliferation [10,11]. Binding of IL-2 to its receptor (IL-2R) leads to a positive feedback by increasing IL-2R expression levels [12]. Additionally, the IL-2:IL-2R complex undergoes rapid internalization and degradation [13] leading to consumption of IL-2 from the intercellular space (Figure 1B). Since this system has been extensively studied and many of its kinetic parameters are known, it can be readily modeled mathematically [14-17]. Recently, IL-2 mediated interactions between T regulatory cells (Tregs) and T effector cells has been studied, showing how depletion of the shared cytokine by Tregs contributes to suppression of effector cells [16,17]. Extracellular feedback driven by IL-2 can also affect polyclonal interactions between effector T cells of different antigen specificities, which interact during their priming within lymphoid organs. These interactions between T cells, together with other factors such as availability of co-stimulatory signals, can shape the repertoire of responding T cells through inter-clonal cooperation and competition [18].

Experimentally, competition between cells was observed as a possible outcome of polyclonal interactions, where activation of cells with one antigen specificity inhibits the response of cells with a different specificity in their vicinity [18]. However, cooperation between polyclonal cells, in which activation of cells is stronger in the presence of neighboring cells with a different specificity, has also been observed in similar experimental models [19]. In a recent report, both cooperation and competition were observed in the same experimental model, which evaluated interactions between groups of endogenous T cells responding to different mixtures of peptides [20]. Furthermore, time dependency of the outcome has also been observed – a limited window of opportunity of a few hours exists for the inhibition of competing cells, both by regulatory T cells [21] and by competing effector T cells [22]. The mechanism underlying this time dependency remains obscure.

Here, we present a mathematical model which suggests that extracellular feedback can account for opposite outcomes: competition or cooperation between interacting polyclonal T cells. The outcome in any specific case depends on quantitative parameters of the activation of the interacting cells, such as their relative TCR stimulation strength. Furthermore, time dependency of the interaction outcome emerges from our model. Thus, if interacting cells are activated at different times, their fate will depend on their relative times of activation. We show that these rich behaviors of the system require non-linearity of the feedback. A linear feedback results in competition and exclusion, where the stronger cell wins regardless of activation time. We propose that this time dependency can serve to balance speed and accuracy of the polyclonal T cell response.

## Methods

We model the behavior of a system comprising two CD4^+^ T cells that have been activated by their cognate antigens, but before proliferation takes place (within ~30 hours after activation) (Figure 1B). As we aim to understand general design principles, we study a simplified model of the system, which still capture its core features. In the following, we describe the main model we use while various modifications are further discussed later and in the Additional file 1.

Figure 1B schematically presents the components of the model. We assume that IL-2Rs are initially expressed at a low constitutive rate, *g*_*c*_, and are degraded at a rate *a*_*U*_, resulting in a low initial number of IL-2R molecules on the T-cell surface (*g*_*c*_*/a*_*U*_ ~ 150, see Additional file 1: Table S1). This provides the cells with an initial responsiveness to IL-2. Upon TCR binding, the T-cells start to secrete IL-2 at a rate *s* that is proportional to the strength of the TCR signal, which is denoted by the parameter 0 *≤ m ≤* 1. The dependence of IL-2 secretion rate on TCR signal strength can be relaxed: assuming that cells secrete IL-2 in a constant rate, regardless of their TCR strength, does not change the general behavior of the model (Additional file 1: Figure S2A). Signaling induced by binding of IL-2 to its receptor results in increased expression of IL-2Rs on the cell surface, thus closing the positive feedback loop. We assume that the induced expression rate of IL-2R is also proportional to the strength of the TCR signal (see below). Additionally, the IL-2R:IL-2 complex is internalized and degraded at a rate *a*_*B*_.

We describe the state of each cell by the number of unbound and bound IL-2R molecules on its surface, *U*_*i*_ and *B*_*i*_, respectively (*i* = 1,2 for the two cells). The free IL-2 in the volume surrounding the cells is denoted by *I*. Following the above assumptions, the dynamics of the free IL-2 is described by the following equation:

(1) I·=s⋅m1+m2−dII−kon⋅I⋅U1+U2+koffB1+B2

and the dynamics of each cell’s state is described by:

(2) $${\overset{\cdot }{B}}_{i}=k_{\mathrm{on}}\cdot I\cdot U_{i}-B_{i}\left(k_{\mathrm{off}}+a_{B}\right)$$

(3) $${\overset{\cdot }{U}}_{i}=g\left(m_{i},B_{i}\right)-a_{U}\cdot U_{i}-k_{\mathrm{on}}\cdot I\cdot U_{i}+k_{\mathrm{off}}\cdot B_{i}$$

where:

(4) $$g\left(m_{i},B_{i}\right)=g_{c}+m_{i}\cdot g_{f}\frac{B_{i}^{2}}{B_{i}^{2}+{K_{f}}^{2}}$$

The TCR signaling strength, *m,* is parameterized to be between zero and one. This value reflects the effect of TCR signaling on the production rate of IL-2R and IL-2. TCR signaling strength mainly depends on the affinity of the antigen to the TCR, and on the number of bound TCRs on the T cell surface. The mapping of these factors into the value of *m* may differ between different systems (mode of stimulation, type of antigen) and can be evaluated experimentally for each system, as we discuss below. However, this mapping does not affect the behavior of the model (Eq. 1-4) describing dynamics of the IL-2-IL-2R system. Co-stimulation is modeled effectively within the TCR signaling strength parameter *m*. Other rate constants that determine the system’s behavior are: *k*_*on*_, *k*_*off*_ - binding and unbinding rates, respectively, of IL-2 to its receptor; and *d*_*I*_, IL-2 degradation rate (or its effective removal from the interaction volume due to transport). As noted before (16,17) IL-2 removal is dominated by its endosomal degradation following binding to its receptor and internalization of the complex, which is modeled by the term $-B_{i}\cdot a_{B}$ in Eq. 2.

IL-2R production rate following TCR induction is given by the positive feedback term in Eq. 4, *m*·*g*_*f*_·(*B*^2^*/*(*B*^2^ + *K*_*f*_^2^), with a maximal synthesis rate of *m*·*g*_*f*_ and half saturation at *B = K*_*f*_. We model dependence of IL-2R production rate on both *m* (TCR signal) and *B* (IL-2 signal), as both signals are required for efficient activation of the IL-2Rα gene [11]. IL-2 mediated feedback on receptor production is assumed to be in the form of a Hill function, with a Hill coefficient of 2. Cooperation is assumed as the main signaling molecule downstream of IL-2R, STAT5, forms dimers after its phosphorylation, which serve as a transcription factor driving expression of the IL-2Rα gene [23]. The values used for these constants in the following simulations are given in Additional file 1: Table S1. We model interaction between cells that are in a close proximity to each other – intercellular distance of the order of 100 microns (e.g. within the same cluster on a dendritic cell [24]). We assume a well-mixed microenvironment, hence a volume of 100 microns^3^ is used in the numerical simulations. Our results are not sensitive to this value (See sensitivity analysis in the Additional file 1).

## Results

### Modeling the IL2 extracellular feedback system

**Figure 2:**
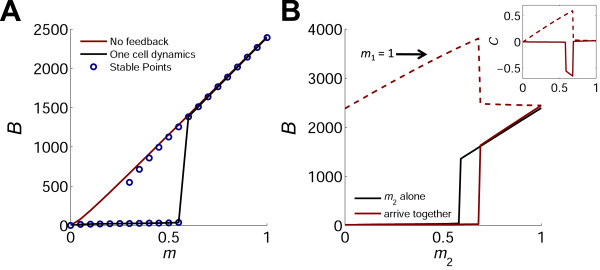
**Activation curves of one cell and of two interacting cells. **(**A**) A single cell. The stable fixed points for the level of bound IL-2 receptor, *B*, were analytically calculated as a function of the TCR signal strength, *m* (blue circles). The system has two branches of stable solutions in an intermediate range of TCR signal strengths. The actual state of the system as calculated numerically (black line) exhibits a sharp transition between low and high levels of *B* when the TCR signal strength exceeds a threshold *m* value. Also shown is the response in the case of no feedback, where the induced synthesis rate of IL-2 receptors is constant (red line). In this case, the number of bound receptors increases linearly with *m* for almost all *m* values, and no threshold exists. (**B**) Two interacting cells. Red line: fixed points of *B* were calculated for a cell that is interacting with a strongly activated cell (*m*_1_ = 1). The black line is the same as in A (for one cell alone), shown for comparison. Due to the intercellular interaction, the activation curve of *Cell2* shifts to the right and the threshold *m* level increases. The dashed red line shows the number of bound receptors of *Cell1* which increases since it can utilize the IL-2 secreted by *Cell2*. *Inset*: The normalized interaction index, *C* (see text and Eq. 5) of the two cells. In the range where *C*_2_ = 0, *Cell2* (black line) is indifferent to the presence of *Cell1*. Where *C*_2_*<* 0, *Cell2* suffers from the interaction (competition and exclusion). In the range where *C*_1_*>* 0, *Cell1* (dashed red line) benefits from the interaction (cooperation).

We analyze the different phases of this dynamic system by calculating its behavior for all activation levels of the interacting cells. We begin by analyzing the response of a single cell to TCR stimulation. In this case, the fixed points of the system can be calculated analytically. The resulting steady state solutions for *B* as a function of TCR stimulation strength, *m*, are shown in Figure 2A. For low values of *m*, there is only one stable solution for *B* which is near its constitutive initial value of *g*_*c*_*/a*_*U*_. In a regime of intermediate *m* values, two stable solutions exist. The level of *B* at the higher stable state increases almost linearly with *m,* while that at the lower state remains low. At high values of *m*, only the higher state remains stable. The actual steady state of the system depends on initial conditions. A solution for the case of *U*_*(t=0)*_ = *g*_*c*_*/a*_*U*_, *B*_*(t=0)*_ = *I*_*(t=0)*_ = 0, is shown in Figure 2A (black curve) and exhibits a sharp transition between low and high levels of *B* as the TCR signal strength is above a critical value, *m* > *m*_*c*_. This critical value is approximated by : $m_{c}\approx \frac{a_{B}{K_{f}}^{2}}{g_{f}}\left(\frac{a_{U}}{g_{c}}\right)$, which gives *m*_*c*_ ≈ 0.5 for the parameter values used in the simulations (see Additional file 1 for derivation). A typical time trace is given in Additional file 1: Figure S16A. This one-cell case of extra-cellular positive feedback shows similar bistability as in the case of intracellular non-linear positive feedback. Bistability in IL-2R level has been observed experimentally [17].

### Extracellular feedback translates quantitative differences in initial conditions into opposite qualitative behaviors: cooperation, coexistence and competition

Next, we solve Eqs. 1,-4, and find the steady state solutions for the case of two interacting T-cells that are activated with TCR signal strengths *m*_1_ and *m*_2_, respectively. Figure 2B shows the fixed points of *B* as a function of *m*_2_ for two cases: a) *Cell2* is activated alone (similar to Figure 2A) and b) the cell is activated alongside a strongly activated cell (*m*_1_ = 1). The threshold of commitment for *Cell2* (the critical *m* value at which *B* increases to its high level), is shifted to higher *m* values in the presence of the strongly activated cell. Hence, at a range of *m* values above threshold, the presence of the strong cell excludes *Cell2* from committing due to competition on IL-2 between the two cells.

To facilitate analysis of such interaction dependent effects, we introduce a normalized interaction index, *C*, describing the relative change in *B* level upon interaction:

(5) $$C_{i}=\left({B_{i}}^{\text{together}}\left(m_{1},m_{2}\right)-{B_{i}}^{\text{alone}}\left(m_{i}\right)\right)/\text{max}\left({B_{i}}^{\text{alone}}\right)$$

with *i* = 1 or 2 for the two cells. This interaction index confers the difference between the cell’s bound IL-2R level if activated alongside another cell or if activated alone. The inset in Figure 2B shows the calculated *C* for the aforementioned scenario. The value of *C* provides a useful description for the outcome of the two cells interaction: *C >* 0 implies one cell benefits from the interaction by utilizing the IL-2 secreted by the other cell – we term this “***cooperation***”, while *C* < 0, implies one cell suffers from the interaction due to IL-2 consumption by the other cell – we term this “***competition***”. When *C* ~ 0, the presence of one cell does not significantly affect the other cell – we term this outcome “***coexistence***”. We note that these definitions are used here relative to a specific cell and are not exclusive; for example, under some conditions both cells can benefit from the interaction (*C*_1_ > 0 and *C*_2_ > 0), while under other conditions one cell may benefit on the expanse of the other (*C*_1_ > 0, *C*_2_ < 0).

**Figure 3:**
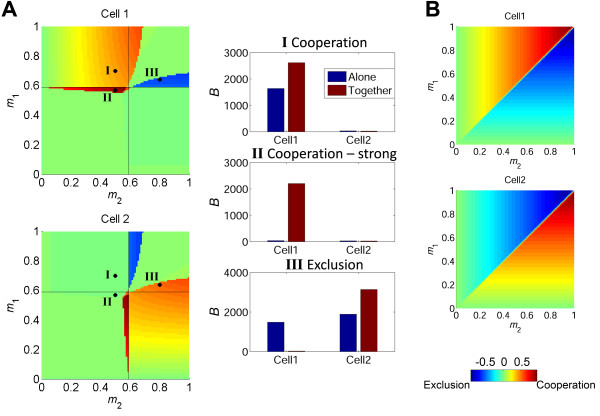
**Extracellular non-linear positive feedback gives rise to cooperation and competition. **(**A**) Left panel shows heat maps of the simulated normalized interaction index, *C*, for the entire range of activation signals (*m*_1_*, m*_2_) of two interacting cells. Color bar on the bottom right depicts *C* values. Different areas emerge, in which the cells cooperate or compete with each other over IL-2. The black lines show the threshold of activation for each cell if it was activated alone. Three representative scenarios are marked with a black dot and the corresponding *B* levels of the two interacting cells are shown in the right panel (red bars), compared to B levels when the cells are activated alone (blue bars). (I) *Cell1*, that would have been activated had it been alone, is activated alongside *Cell2* which would not have passed the activation threshold had it been alone. In this case, the stronger cell utilizes the IL-2 of the weaker cell and increases its number of bound IL-2R. (II) Two cells that would not have been activated were they alone pool their IL-2 and as a result *Cell1* that is activated more strongly is driven above threshold. (III) Two cells that are above threshold by their own are activated together. In this case, *Cell2* that is activated more strongly depletes the IL-2 reservoir and thus pushes its neighbor beneath the activation threshold. (**B**) Simulated *C* values for two interacting cells in the case of a (hypothetical) linear feedback. In this case the interaction would *always* lead to mutual exclusion, resulting in the activation of the stronger cell (same color bar as in A).

Plotting *C* for all possible *m*_1_ and *m*_2_ values reveals a complex interaction map, in which various outcomes of the interaction exist as a function of the relative activation strength of the two cells (Figure 3A). We also present in this figure the steady-state levels of bound IL-2R of the two cells with and without interaction, for three representative cases identified by points shown on the map. Point I represents cooperation, where *Cell1* that is already above commitment threshold, benefits from the presence of a sub-threshold *Cell2*, and consequently obtains higher levels of *B*. Point II represents a case of stronger cooperation, whereby *Cell1* is originally below its commitment threshold, but becomes strongly committed in the presence of a sub-threshold *Cell2*. In this case, the interaction results in a committed cell, whereas without interaction both cells would not have been committed. An opposite outcome is seen in point III, where there is competitive exclusion of one cell by another, even though both cells are above their commitment threshold. The stronger *Cell2*, with a higher level of activation (*m*_2_ *> m*_1_), depletes IL-2 and prevents commitment of *Cell1*. In this case of profitable inhibition, the stronger *Cell2* benefits from the interaction and reaches a higher level of *B* in the presence of *Cell1* than it would have reached on its own. Note that when activation of both cells is strong enough, the interaction has a minimal effect on both cells (coexistence - green region in Figure 3A, upper right quadrant). Thus, this simple system of two interacting cells, in the presence of extracellular positive feedback on receptor levels, manifests a complex behavior. Relatively small *quantitative* changes in the activation parameters lead to *qualitatively* opposite outcomes for the interacting cells.

A non-linear feedback is essential for obtaining these complex system behaviors. We consider two hypothetical cases: a) no IL-2 mediated feedback on IL-2R synthesis rate and b) a linear feedback on IL-2R synthesis rate. In both cases, the number of bound receptors increases linearly with *m* for almost all values of *m*, and no bistability occurs (see Additional file 1 for a detailed analysis). In the first case, receptor synthesis rate depends only on *m*, and not on the level of bound receptors, and is given by *m*·*g*_*i*_. In this case, cells always slightly benefit from the interaction (Additional file 1: Figure S1). In the second case, receptor synthesis rate is given by: *m*·*g*_*i*_·*B.* As a result, when two cells interact they do not cooperate but always compete, with the stronger cell excluding the inferior one (Figure 3B).

Our results are in general not sensitive to the exact values of parameters used in the simulations (see sensitivity analysis in the Additional file 1: Figures S3-S9). Changing the rate constants of Additional file 1: Table S1 mainly affects the single cell behavior, by modifying the threshold value for *m*. Around the modified single-cell threshold, the two cell interaction space does not change significantly, and shows regions of cooperation, competition and coexistence in similar positions relative to the threshold values of the two cells. We also checked the sensitivity of these results to some of the assumption made in our model. One assumption is that IL-2 is made only by the two interacting cell, and is dependent on *m* (Eq. 1). We modified this assumption in two ways: first, we checked a case in which cells secrete IL-2 at some constant rate, independent of the level of *m*. Second, we added a constant production term of IL-2, presumably made by other nearby cells and entering the interaction volume of the two modeled cells. Both perturbations didn’t change the qualitative behavior of the model, which still exhibits regions of cooperation, competition and coexistence (Additional file 1: Figure S2A, B).

### Interaction outcome depends on the relative activation times of the two cells

**Figure 4:**
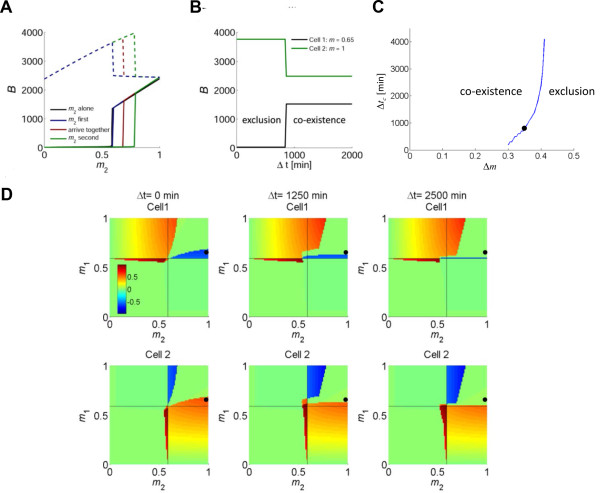
**Interaction outcome depends on the relative time of activation of the two cells. **(**A**) The activation curve of a T-cell with varying *m*_2_ values that is activated before, alongside or after a strong T-cell, *m*_1_ = 1. The black line marks the activation curve for a single cell alone. The threshold *m*_2_ level depends on the order of activation. If *Cell2* is activated markedly (2500 minutes) before *Cell1*, its activation curve does not change significantly (blue). However, if the two cells are activated together the activation curve shifts to the right and the threshold *m* value for *Cell2* increases (red, similar to Figure 2B). Thirdly, if the order reverses and *Cell1* is activated a long time (2500 minutes) before *Cell2*, the competition is increased and the activation threshold for *Cell2* is shifted to even higher values. (**B**) Time dependent competition in a representative case (*m*_1_ = 0.65 and *m*_2_ = 1). *Cell1* is activated at time 0, and after Δt minutes, *Cell2* is activated. A critical time window for competition exists: if *Cell2* (green line) is activated within the time window, it excludes the weaker *Cell1* (black line). However, if *Cell2* is activated after the window closes, it is unable to suppress *Cell1*, and the two coexist. (**C**) The length of the critical time window increases with the difference in activation strengths of the two cells. The line represent the calculated time window for exclusion, for the case of a cell with *m*_1_ = 0.65 activated at time 0, interacting with a stronger cell (*m*_2_ = *m*_1_*+* Δ*m*) that is activated later. The scenario shown in (B) is marked by a black dot. (**D**) Simulated normalized interaction index, C, of two interacting cells, for 3 different time delays: *Cell2* is activated with a delay of Δt = 0, 1250, 2500 minutes after *Cell1.* Color bar is the same for all panels. Note changes in both competition and cooperation behaviors over time. For example, when *Cell1* is above threshold, the ability of a stronger *Cell2* to exclude it diminishes with time (top row, blue patch in upper right quadrant). The black dot marks the case of *m*_1_ = 0.65 and *m*_2_ = 1 that is illustrated in (B).

Our model reveals another interesting feature mediated by extracellular positive feedback: the outcome of the intercellular interaction depends on the relative time of activation (Δt) of the cells. To illustrate this, consider a situation in which *Cell1* is strongly activated (*m*_1_ = 1). As described above, the commitment threshold of *Cell2* is shifted towards higher *m* values in the presence of the other cell, thus it will need a stronger TCR stimulation in order to exceed its threshold. However, this is the case only if the two cells are activated at the same time (Figure 4A, solid red line). If *Cell2* is activated long enough before *Cell1*, this competition effect disappears, and the commitment threshold of *Cell2* does not change significantly relative to its threshold level when alone (Figure 4A, solid blue line). The opposite happens if the stronger *Cell1* is activated long enough before *Cell2*. In this case, the competition effect is even more pronounced than when the cells are activated together (Figure 4A, solid green line).

The temporal transition point between outcomes of the interaction lies at a critical relative time of activation, Δt_c_, which depends on the TCR stimulation strength of both cells. In Figure 4B, we show a specific example for the time window for competition, for two interacting cells with *m*_1_ = 0.65 and *m*_2_ = 1. If the stronger *Cell2* is activated within the critical time window after the activation of *Cell1* (Δt < Δt_c_), it competes with and excludes the inferior *Cell1*. However, if *Cell2* is activated after the window has closed (Δt > Δt_c_), it cannot exclude *Cell1* and both cells coexist. For this case, Δt_c_ ≈ 14 hours, which is similar to the critical time window for competition between effector T cells of different TCR specificities that was observed experimentally [21,22]. Our analysis shows that the length of this critical time window increases as the difference in activation strengths of the two cells is larger (Figure 4C). Hence, the stronger the later activating cell, the more time it has to exclude its inferior competitor.

The overall time dependence of the system over the entire interaction phase-space is shown in Figure 4D, for cases in which cells are activated together (left) or in which *Cell1* is activated before *Cell2* (middle: Δt = 1250 minutes; right: Δt = 2500 minutes). Activation timing affects the system’s behavior mainly in the region where both cells are above their commitment threshold (upper right quadrant). If *m*_1_ *> m*_2_, pre-activation of *Cell1* leads to its dominance together with inhibition of *Cell2*, in a range of *m* values which increases with the activation time difference. Conversely, if *m*_1_ *< m*_2_, the region of dominance of *Cell2* shrinks the later it is activated. However, if both cells are below commitment threshold (lower left quadrant), the later activation of *Cell2* allows for its bystander commitment in a somewhat larger parameter range, as more IL-2 accumulates due to the pre-activation of the sub-threshold *Cell1*. These effects of time of activation do not occur if there is no feedback or if the feedback is linear. In those cases, the cell with higher *m* value wins and excludes the other cell regardless of the relative activation time of the two cells (see Additional file 1 for detailed analysis).

A specific scenario is the interaction between cells with the same *m* value. When the cells are activated at the same time, interaction between two cells that are slightly below commitment threshold leads to their commitment (See Additional file 1). In this case, both cells profit from the increased level of IL-2 provided by the larger cell concentration, akin to quorum sensing. A more complex behavior emerges when the two cells are activated at different times (See Additional file 1: Figure S14 for a detailed analysis). If the *m* value of both cells is slightly below threshold, the later activated T-cell can utilize the IL-2 secreted by the earlier activated T-cell and consequently become committed while the first remains uncommitted. If the cells’ *m* value is above threshold, there is a competition between the T-cells that could lead to exclusion of either the earlier cell (for *m* values slightly above threshold) or the later cell (for intermediate *m* values). This example emphasizes our previous results showing that under non-linear extracellular positive feedback, intercellular interactions can lead to non-trivial behaviors, which depend strongly on the timing of activation.

Time dependence was observed also for suppression of effector T cells by Tregs [21]. We simulate this situation using our model showing explicitly how suppression by Tregs is modulated by relative activation time for all combinations of activation levels of the two cells (Additional file 1: Figure S15).

### Multicellular interactions with a larger number of cells

So far, we have considered interaction between two cells. Yet, the features we have demonstrated are more general and not limited to the two cell case. As an example, we study the interaction between an ensemble of 9 cells with uniformly distributed *m* values in the range 0–1 and a cell with *m* = 1, denoted as *Cell1*. We calculate the steady-state level of *Cell1*’s bound IL-2R for a large number of cases where the *m* values of the other 9 interacting cells are randomly drawn from the uniform distribution. To study the effects of timing, we examine a case in which all the cells are activated together and a case in which *Cell1* is activated with a time delay.

**Figure 5:**
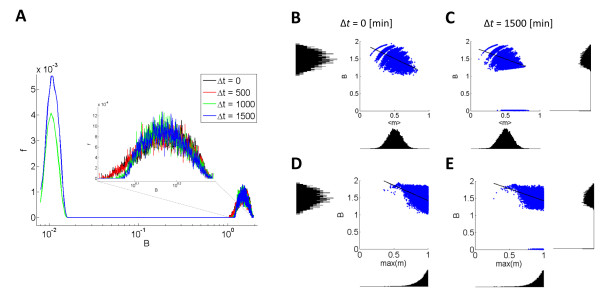
**Interaction between multiple cells. **(**A**) The distribution of bound IL-2R, *B*, of a cell with *m* = 1, *Cell1*, while interacting with 9 other cells with random *m* values. The number of IL-2R of *Cell1* was normalized by its value when there is no interaction. Δt is the time difference between the activation of *Cell1* and the random ensemble. When the time delay is short (black, red), *Cell1* benefits from the interaction. However, when *Cell1* is activated after a longer delay (green, blue), a bi-modal distribution emerges with an additional peak around very low values of *B*. Even the ‘strongest’ cell can be excluded if activated too late. The volume of interaction for this graph is 5 times the volume that was used for 2 cell interaction and thus the cell density is the same. The *m* values in each ensemble were randomly pulled out of a uniform distribution. Each distribution is the result of 10,000 runs. (**B-E**) The effect of ensemble composition on the response of *Cell1*. As the mean TCR strength of the competitors is larger (B, C) the amount of IL-2R of *cell1* decreases. If the delay is long, exclusion may occur only if the mean *m* of the competing group is high enough (above ~ 0.5). On the top of it, the maximal competitor has to be strong enough (above ~ 0.75) for exclusion to occur (D, E).

Figure 5A shows the probability distribution of the level of bound IL-2R of *Cell1* normalized by its value when there is no interaction. If the cells are activated together or when the delay is short (500 minutes), in most cases *Cell1* benefits from the interaction by a factor of ~1.5. However, when the delay is longer, *Cell1* can be excluded by its competitors and the probability distribution exhibits a peak around zero which has not been observed in the former case. Moreover, the probability of being excluded is increased as the time delay is longer.

The composition of the competing ensemble affects the response of *Cell1*. As the mean TCR strength of the competitors is larger (Figure 5B, 5C) the level of IL-2R on *Cell1* decreases. When the delay is long, exclusion may occur only if the mean *m* of the competing group is high enough (above ~ 0.5). Another condition for exclusion is that the strongest competitor is strong enough (above ~ 0.75) (Figure 5D, 5E). Thus, although *Cell1* has been activated as strongly as possible, it could be excluded by an ensemble of inferior cells if it is activated too late.

## Discussion

Recent work [16,17] described IL-2 mediated suppression of effector T cells (Teff) by T regulatory cells (Treg), showing the conditions under which competition for IL-2 serves as an efficient suppression mechanism. Our model examines the effect of IL-2 mediated extracellular feedback on the interaction between effector T cells during their activation. While competition is the prominent case in Teff-Treg interactions, it is only one of the scenarios that Teff-Teff interactions can give rise to. In this polyclonal interactions scenario, both cells secrete IL-2, and thus both cells can also benefit from the IL-2 that their neighbors secrete. While the equations regarding kinetics of IL-2 and its receptor are naturally similar, there are a number of significant differences. In particular, a Treg-Teff interaction is non-symmetric, as Tregs express an initial high level of CD25, and do not secrete IL-2. We study the symmetric case of interaction between two effector cells, where both cells are governed by the same equations and the only difference is in the strength of TCR signal. This situation models the abundant case of polyclonal interactions that occur in vivo between T cells with different TCR specificities. These interactions play an important part in shaping T cell responses through inter-clonal interactions. Our model presents a first mathematical/computational attempt to study the role of IL-2 extracellular feedback in this context.

The significance of examining such a scenario is clear from a biological vantage point, as this type of interaction naturally occurs during T cell activation, when cells with different TCR specificities are activated within the same microenvironment, e.g. surrounding the same antigen presenting cell [24]. In effect, many T cell interactions are polyclonal Teff-Teff interactions – most T cell mediated immune responses start with polyclonal T cell activation. Thus, it is important to understand T cell interactions within this context, as it shapes the repertoire of the immune response.

### T cell interactions mediated by IL-2 extracellular feedback can result in opposite outcomes: competition or cooperation

We show that intercellular interactions mediated by IL-2 and amplified by the extracellular positive feedback on IL-2R levels can account for both competition and cooperation between activated T cells. Experimental evidence for competition and cooperation between T cells has been available for several decades [25,26], and a role for IL-2 in mediating competition has been suggested [27]. There are other factors that influence polyclonal T cell interactions at early stages of activation, such as competition over peptide-MHC (pMHC) complexes, limited co-stimulation by the antigen-presenting cell (APC) or limited physical access of T-cells to the APC [18]. However, several experiments have demonstrated competition and cooperation in setups lacking these factors [20,22]. Consequently, it has been suggested that secreted factors such as cytokines can play a role in driving intercellular competition and cooperation. Furthermore, both cooperation [19] and competition [22] were observed in experiments in which T cells of two TCR transgenic populations, with different specificities, were transferred into recipient mice and were activated by corresponding cognate peptides. Use of two different antigens presented by different MHC molecules ensured that competition was not over pMHC complexes. Though the two systems are different in some of the experimental details, one may still wonder under what conditions either outcome, cooperation or competition, is observed.

An even more direct scenario was described in a recent report which evaluated interactions between endogenous T cells responding to different mixtures of peptides [20]. T cells responding to weakly binding pMHC complexes were inhibited in the presence of competitive T cells, whereas T cells responding to strongly binding complexes gained from the presence of the same competing cells [20]. This demonstrates that even within the same experimental setup, opposite qualitative outcomes can be reached, by changing quantitative parameters of the T cells’ activation. Our model is in line with these experimental observations, as it predicts that in the presence of IL-2 extracellular feedback, strongly activated cells will benefit from interactions with average competitors, while cells that are weakly activated will suffer from such interaction. Thus, our model can resolve the seemingly contradicting experimental outcomes demonstrated for T cell interactions in those experiments, by providing a mathematical description for the complex relationship between relative TCR activation strength of the interacting cells and outcome of the interaction. Such inter-clonal interactions are of importance in many sub-fields of immunology, including vaccine design, cancer immunology, allergy and others.

Recently, multiphoton microscopy experiments have demonstrated that there are three stages in the process of in-vivo T cell activation and commitment to proliferation. In the first stage, T cells that arrive asynchronously into the lymph node scan residing APCs for their target peptide. If a cognate peptide is found the T cells undergo activation, stop at the APC and stay within the lymph node. In the last stage, T cells undergo proliferation and migrate out of the lymph node. Thus, at the second stage T cells arrest for 10–20 hours on the presenting APC [24,28-30] and secrete IL-2 to the cell’s microenvironment [31]. In addition, T cells form clusters around APCs at this stage, facilitating intercellular interactions among T cells. Thus, this might be the stage in which the interaction we model takes place, between T-cells activated by the same or by a nearby APC.

Our model provides some predictions regarding the roles of IL-2 extracellular feedback in determining the outcome of inter-clonal T cell interactions, which can be tested experimentally. One possibility is to use an in vitro culture system in which T cells of two different TCR specificities will be co-cultured and allowed to interact. For example, T cells from two different strains of TCR transgenic mice can be co-cultured and activated by dendritic cells that present both cognate antigens. Such a system provides a controlled setup for varying parameters of the interaction and measuring their effect. One prediction of the model is that addition of external IL-2 to the growth medium will decrease competition, while depletion of IL-2 can enhance the range of competition between the two cell types. Using such a system it can also be possible to directly test model predictions regarding the effect of time of activation, as one type of cells can be introduced into the culture at later time points. Model predictions can also be tested in vivo, for example by injecting IL-2 to increase its availability during the response in the mouse models used in [19,20,22]. A similar approach was recently used in [16] to reduce suppression by regulatory T cells. Our model predicts that excessive levels of IL-2 will reduce inter-clonal competition in those in vivo systems.

### Extracellular feedback can balance speed and accuracy

Our model further predicts that the outcome of interactions depends on the relative activation time of the interacting cells. Thus, a stronger cell will exclude its inferior competitor if it is activated either before or alongside with it, but it will coexist with the latter if it is activated too late. Time dependency of intercellular interactions has been observed experimentally. Competition between T cells of different specificities was observed in-vivo only when the competing cells were introduced to the animal within a time window of several hours post introduction of the first T cell population, otherwise the two populations coexisted [22].

We suggest that such time dependency may serve to balance two opposite needs of early T cell responses: the need to employ the strongest reaction, and the need to act swiftly against a proliferating pathogen. Employing the strongest reaction in a polyclonal system is a matter of accuracy; that is choosing the clone (or small set of clones) that can best recognize the pathogen. T cells with random TCR specificities continuously scan the body in search of antigens, entering and leaving lymph nodes. A T cell that has engaged its cognate antigen will arrest on the APC (as mentioned above). This offers an opportunity to wait for other T cells, in case a better cell arrives, one that has a higher affinity to the same or related antigens. It can be beneficial to allow the stronger cell to exclude the weaker cell, in a way that will lead to affinity maturation of the T cell response. However, this waiting time should be limited in order to exert the immune response as quickly as possible. Our model shows how extracellular feedback can allow for balancing speed versus accuracy of early T cell responses, by providing a limited window of opportunity for cells to compete. We show that this window of opportunity is shorter the stronger the TCR engagement: thus a more suitable cell allows less time for other cells to compete with it, and it is activated faster than less suitable cells. Conversely, a less suitable cell will wait longer for a better cell to arrive (Figure 4C).

**Figure 6:**
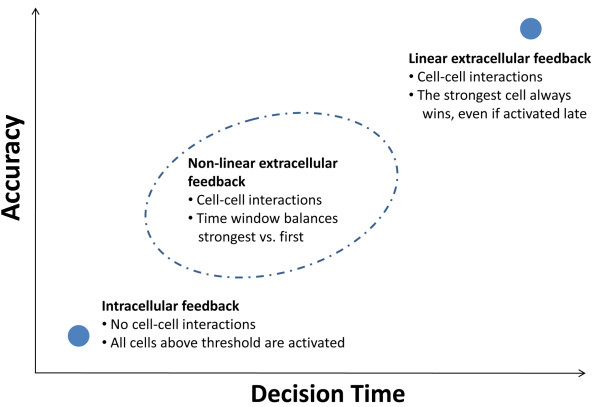
**Non linear extracellular feedback: a design principle that can balance speed and accuracy of a recognition system.** Consider a system of cells that can be activated by recognition of a ligand. Each cell responds at a different level, depending on its specificity, and recognition threshold for cell activation is set through a positive feedback. Cells search for ligand and are thus activated in a random timing. We compare three cases: an intracellular feedback, a linear extracellular feedback and a non-linear extracellular feedback. In the case of an intracellular feedback (no intercellular communication), the system’s response is fast as every cell that passes the recognition threshold is activated. However, the response will be less specific, since all cells above threshold respond, not only the strongest ones (lower left point). In the case of a linear extracellular feedback, the strongest cell always wins, regardless of the time of its activation. In this case specificity of the system’s response is high, but time is lost until the strongest cell is activated, thus the response can be slow (upper right point). A non-linear extracellular feedback balances this tradeoff by opening a window of opportunity for competition. The strongest cell that arrives during this time window will win. The length of this time window is tunable, and depends on the relative activation levels of the interacting cells.

We propose extracellular non linear feedback as a general design principle that allows a recognition system to balance between accuracy and decision time (Figure 6). If there is no communication between cells, such as when the feedback is *intracellular*, the only factor that plays a part in the decision is whether or not a cell has passed its threshold (which depends on the properties of the feedback). In this case, the system’s response is fast since it does not wait for the strongest responder. Accuracy, however, is low as many cells within a large range of specificities will respond. The other extreme is when communication exists between cells, via a *linear extracellular* feedback (see Additional file 1). In this case, the strongest responder always wins regardless of its time of activation. Thus, the system’s specificity is high however its response is slower. By having a *non-linear extracellular* feedback, cells will wait for a stronger cell only for a limited time (Figures 4, 5, 6). This tunable design principle allows the system to balance the speed of the response and its accuracy. A similar notion has been presented in a recent review about collective decision making in animal groups [32].

### Modeling considerations

We use a simplified model of the system, aiming to gain understanding of general properties of the extracellular positive feedback loop. Thus, we do not explicitly model STAT5 and its phosphorylation, assuming that the level of pSTAT5 is in quasi-equilibrium with the level of *B*, such that the receptor synthesis rate directly depends on the level of *B*. In addition, the IL-2R has three subunits (α,β,γ). Feedback by IL-2 only enhances production of the α subunit. As this subunit is expressed at a much higher level on the surface of activated T cells compared to the other two subunits, it is assumed to govern the cellular response to IL-2. We find that as long as the level of the IL-2Rβ subunit does not change considerably, the system can be accurately described by our simplified model, and the rate constants of a more detailed model, that includes all 3 receptor subunits, are close to those used in our simulations (see Additional file 1: Figures S12 and S13). Our simplified approach allows for a more intuitive understanding of the general properties of the IL-2 feedback system, and for generalization to other systems of extracellular positive feedback.

Other factors can also influence the outcome of the interaction, for example different initial levels of IL-2 receptors expressed by the cells, or other differences in the signaling capacity of the IL-2 and TCR signaling pathways [33]. Such differences can result from stochasticity in gene expression [34,35]. As a demonstration of these effects, we present in Additional file 1: Figure S18 the results of a simulation of our model for the three cases shown in Figure 3A, but in which the initial number of receptors expressed by the interacting cells is noisy. The two cases showing cooperation (cases I, II) are not sensitive to this change in initial conditions, and the stochastic results are the same as the deterministic ones. However, for the case of competition and exclusion (case III), introduction of noise leads to a bi-modal distribution, where the deterministic results are obtained in ~70% of the cases, while in the rest there is no exclusion. Our current deterministic model can be extended into a stochastic model to account for such differences between cells that result from stochasticity, for example by using the Gillespie algorithm [36] to model all the reactions.

We show that the non-linearity of the feedback is crucial for obtaining a wide range of possible outcomes for intercellular interactions. A linear feedback would result in a “winner-takes-all” scenario, where the cell with the higher level of TCR signal will out-compete and exclude its inferior neighbor, regardless of the differences in their activation levels or their activation times. This scenario is similar to that studied in population dynamics of two populations competing for a shared resource [2].

Our results are obtained under the assumption of a well-mixed environment, implying that a cell secreting IL-2 does not have a preference in using it compared with its neighbor. This issue was discussed before [16], where it is shown that for the specific kinetic values of the IL-2 system, autocrine signaling is not dominant and a well-mixed environment is a good approximation. The actual mode of transport of IL-2 inside lymph nodes has not been established and further experimental investigation is required for gaining a better understanding of the transport properties, which can have important influence on IL-2 mediated intercellular interactions. Our model can be extended to include different modes of IL-2 transport, e.g. diffusion [17], non-isotropic IL-2 secretion [37] and also specific cell-cell geometries such as IL-2 secretion into low volume synapses between clustering T cells, as was recently observed in vitro and in vivo [38].

When relating our model to experimentally measurable parameters, two points should be considered. First, we use a normalized parameter *m* to describe the level of TCR signaling. In practice, TCR signaling level can be varied, for example, by changing antigen concentration or using signaling inhibitors such as cyclosporine. One can relate the experimental variable to *m*, by measuring the levels of IL-2Rα (CD25) for varying levels of TCR stimulation, and using the resulting curve for normalization. To avoid effects of extracellular feedback, this calibration would be preferably done with cells lacking IL-2 secretion (e.g. from IL2 knockout mice), in the presence of a constant level of IL-2. Second, we used *B* (number of bound receptors) as an indicator of cell state. Experimentally, *B* is difficult to measure while related parameters such as the level of pSTAT5 or total receptor level (*B*+*U*) are more accessible. It was recently shown experimentally [16] that the level of *B* is linearly related to the level of pSTAT5, which is the main signaling molecule downstream of the IL2R complex. Thus, *B* is a good estimate for the level of IL2 induced signaling. In Additional file 1: Figure S19 we present the results of our model in terms of the total number of IL2R (*B*+*U*). We note that in contrary to *B*, the total level of receptor does not increase in the region of cooperation (around point I in Figure 3A), due to the different internalization times of the unbound and bound receptor.

### Generality of the model

Finally, we note that similar feedback and consumption mechanisms apply for cytokines other than IL-2. For example, the cytokine IL-4 increases expression levels of its own receptor, while cytokine-bound receptors are internalized and degraded [39]. Thus, our model can be applied also to other cytokine mediated intercellular interactions, such as cytokine-driven differentiation of CD4^+^ cells [40,41], or differentiation of CD4^+^ T cells into memory cells, in which competition for IFNγ has been suggested to play a role [42]. Taken together, our model offers a general framework for studying IL-2 induced commitment to proliferation of T cells as a collective process. This can apply not only to the case of interactions between unprimed cells, but also to interactions that involve effector and regulatory cells (see Additional file 1), or interactions which include memory T cells. The latter have been recently shown to drive bystander commitment of CD4^+^ T cells, potentially involving IL-2 [43,44].

## Conclusions

We suggest a role for extracellular feedback by studying interactions between activated polyclonal T cells as a model system. Using mathematical modeling we find that extracellular feedback can give rise to opposite outcomes of the interaction – either competition or cooperation between the interacting cells, and that this outcome depends on the parameters of the activation. In addition, the interaction outcome depends also on the timing of activation of the cells. As a result, a critical time window exists after which a stronger cell cannot exclude an inferior competitor, as was experimentally observed. We suggest that extracellular feedback can balance a speed vs. accuracy tradeoff in T cell activation – choosing the most suitable responder under the clock of a proliferating threat. These findings can serve to better understand the way the repertoire of an immune response is shaped, in response to pathogens, vaccines and tumor detection.

## Competing interests

The authors declare that they have no competing interests.

## Authors' contributions

YS, NW, YEA, TT and NF designed the study; YS and NW performed the study; YEA contributed to early stages of this work; YS, NW and NF wrote the manuscript. All authors read and approved the final manuscript.

## Authors' information

Co-first authors: Yonatan Savir and Nir Waysbort.

## Supplementary Material

Additional file 1 **Table S1.** The parameters of the model. **Table S2.** Parameters related to the full IL-2R dynamics. **Figure S1.** Phase space diagrams for the case of a constant induced receptor synthesis rate. **Figure S2.** Phase space diagrams for the case of a constant IL-2 secretion rate. **Figure S3.** Variation in the IL-2 secretion rate, *s.***Figure S4.** Variation in the IL-2 degradation rate, *d*. **Figure S5.** Variation in the constitutive receptor synthesis rate, *g*_*c*_ and in the unbound receptor internalization rate, *a*_*U*_. **Figure S6.** Variation in the bound receptor internalization rate, *a*_*B*_. **Figure S7.** Variation in the maximal induced synthesis rate, *g*_*f*_. **Figure S8.** Variation in the feedback threshold level, *K*_*f*_. **Figure S9.** Variation in the Hill coefficient, *n*. **Figure S10.** The competition between *m*_1_ = 1 and *m*_2_ = 0.7. **Figure S11.** Dynamical orbits of the competition between *m* = 1 and *m* = 0.7. **Figure S12.** One cell fixed points results for the full and effective models. **Figure S13.** Phase space diagrams of the full and effective models. **Figure S14.** Two cells with the same *m* value exhibit a complex time dependent behavior. **Figure S15.** Interaction of an effector T cell with a regulatory T cell is time dependent. **Figure S16.** Interaction phase diagrams for an effector T cell that is interacting with a non-secreting T cell. **Figure S17.** Examples of time dynamics. **Figure S18.** The distribution of *B* when the initial number of IL-2R is noisy. **Figure S19.** Behavior of the normalized number of total IL-2R (B + U). Click here for file
