# Supplementary material for: Balancing speed and accuracy of polyclonal T cell activation: a role for extracellular feedback
Source: BMC Syst Biol. 2012 Aug 27;6:111. doi: 10.1186/1752-0509-6-111 (PMC3512529; doi:10.1186/1752-0509-6-111)
Supplement: Additional file 1 — Table S1. The parameters of the model. Table S2. Parameters related to the full IL-2R dynamics. Figure S1. Phase space diagrams for the case of a constant induced receptor synthesis rate. Figure S2. Phase space diagrams for the case of a constant IL-2 secretion rate. Figure S3. Variation in the IL-2 secretion rate, s.Figure S4. Variation in the IL-2 degradation rate, d. Figure S5. Variation in the constitutive receptor synthesis rate, gc and in the unbound receptor internalization rate, aU. Figure S6. Variation in the bound receptor internalization rate, aB. Figure S7. Variation in the maximal induced synthesis rate, gf. Figure S8. Variation in the feedback threshold level, Kf. Figure S9. Variation in the Hill coefficient, n. Figure S10. The competition between m1 = 1 and m2 = 0.7. Figure S11. Dynamical orbits of the competition between m = 1 and m = 0.7. Figure S12. One cell fixed points results for the full and effective models. Figure S13. Phase space diagrams of the full and effective models. Figure S14. Two cells with the same m value exhibit a complex time dependent behavior. Figure S15. Interaction of an effector T cell with a regulatory T cell is time dependent. Figure S16. Interaction phase diagrams for an effector T cell that is interacting with a non-secreting T cell. Figure S17. Examples of time dynamics. Figure S18. The distribution of B when the initial number of IL-2R is noisy. Figure S19. Behavior of the normalized number of total IL-2R (B + U). [file 1752-0509-6-111-S1.pdf]

Supplementary Information

1. Parameters of the model

The dynamics of the system for one cell (no intercellular interactions) is given by:

$$\begin{aligned}
\dot{I} &= s \cdot m - d_I I - k_{on} \cdot I \cdot U + k_{off} B \\
\dot{B} &= k_{on} \cdot I \cdot U - k_{off} \cdot B - a_B \cdot B \\
\dot{U} &= g_c - a_U \cdot U - k_{on} \cdot I \cdot U + k_{off} \cdot B + m \cdot g_f \frac{B^2}{B^2 + K_f^{\,2}}
\end{aligned} \qquad \text{(S1)}$$

I is the amount of free IL-2, *U* and *B* are the amounts of unbound and bound IL-2R, respectively. Table S1 summarizes the parameters of the model. These parameters were used for the model presented in the main text.

Parameter	Description	**Value**	**Comments**
m	TCR signal	(0-1)	m =1 yields maximal IL-2 secretion rate and maximal induced receptor synthesis rate.
s	Maximal IL-2 secretion rate	100 [min^{-1}]	[1, 2]
d	IL2 degradation rate	1e^{-3} [min^{-1}]	An estimate, a similar value was used in [3]
k_{on}	Binding constant	3.1e^{-6}[min^{-1}]	[4] Assuming volume of interaction, V = 1nL
k_{off}	Dissociation rate	0.0138[min^{-1}]	[4]
g_c	Constitutive receptor synthesis rate	1[min^{-1}]	Defined as to give ~100 IL2R's at steady state with no induction [5]
a_U	Unbound receptor internalization rate	0.01[min^{-1}]	
a_B	Bound receptor internalization rate	0.04[min^{-1}]	[6]
g_f	Maximal induced synthesis rate	200[min^{-1}]	Calculated as to give maximal ~15000 IL2R's at steady state with maximal induction
K_f	Feedback threshold	500	

Table S1: The parameters of the model.

Note that the values of *I*, *B* and *U* are in units of numbers of molecules and not concentrations and thus first order rate constants (e.g. *d*) have units of 1/time.

2. A constant induced synthesis rate of receptors

Here, we consider the case in which receptor synthesis rate depends only on m, and not on B, hence is independent of IL2. In this case, we can use Eqs. S1, with $K_f = 0$. In general, the fixed points of (S1) for only one T-cell obey:

$$\begin{aligned} & I = \frac{s \cdot m \cdot \alpha}{U + d \cdot \alpha};\ \ with\ \ K = \frac{a_B + k_{off}}{k_{on}}; \alpha = K / a_B \\ & B = \frac{U \cdot I}{K} = \frac{U \cdot I}{a_B \cdot \alpha} = \frac{m \cdot s \cdot U}{a_B (U + d \cdot \alpha)} \\ & 0 = g_c - a_U U - a_B B + m_i \cdot g_f \frac{B^2}{B^2 + K_f^{\ 2}} \end{aligned} \tag{S2}$$

With $K_f = 0$, Eqs. S2 can be reduced into a quadratic equation for U, which can be solved as:

$$\begin{aligned} & C_1 = g_c + m(g_f - s) - a_U d\alpha \\ & C_2 = 4a_U d\alpha (g_c + mg_f) \\ & U = \frac{C_1 + \sqrt{C_1^{\ 2} + C_2}}{2a_U} \end{aligned} \tag{S3}$$

In the case of strong induction ($mg_f >> g_c$, $U >> d\alpha$), the number of unbound receptors in steady state is $U \approx m(g_f - s)/a_U \approx 10000$ and the number of bound IL2R is $B \approx ms/a_B \approx 2500$. The recognition curve (i.e. bound IL2R a function of m) is linear as long as $U >> d\alpha$ that is when $m >> m_{c1} = (a_U d\alpha - g_c)/(g_f - s)$. For any reasonable parameters, $m_{c1} << 1$ and thus the recognition curve is linear for almost all range of m values (Fig. 2A in the main text). In this case, the T-cells always benefit from the interaction (Fig. S1) and the interaction does not depend on time.

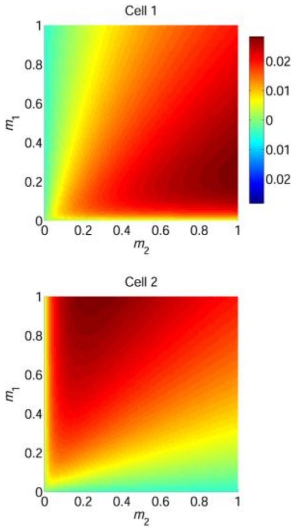

Figure S1: Phase space diagrams for the case of a constant induced receptor synthesis rate. The normalized interaction index, C (as defined in Eq. 5 in the main text) is presented for both interacting cells (color bar). The interaction does not depend on time. The cells always benefits, in a negligible way, from the interaction.

3. A linear induced synthesis rate of receptors

Here, we consider the case in which receptor synthesis rate depends on both m and B. However, the IL2 induced feedback is linear, unlike the non-linear feedback that is discussed in the main text. Thus, the term $m \cdot g_f \cdot (B^2 / (B^2 + K_f^2)$ is replaced with $m \cdot g_f \cdot B$. The fixed points are given by:

$$
\begin{aligned}
C_1 &= g_c \cdot m^2 \cdot s + a_B \cdot (g_c - m \cdot s - a_U d\alpha) \\
C_2 &= 4 \cdot a_U \cdot a_B{}^2 \cdot d \cdot \alpha \cdot g_c \\
U &= \frac{C_1 + \sqrt{C_1{}^2 + C_2}}{2 a_U a_B}
\end{aligned}
\qquad \text{(S4)}
$$

In a similar way to the case of constant synthesis, B is almost always a linear function of m (Fig 2A in the main text). In this case there is *always* mutual exclusion (Fig. 3B in the main text) between interacting T-cells. The winner is the T-cell with the highest m value.

4. A constant secretion rate

In our model, the secretion rate of IL-2 depends on the strength of the TCR activation, m. The overall secretion rate is $s \cdot m$ where s is the maximal secretion rate. However, it is insightful to consider also two variants of the model: 1) The case of constant secretion rate, that is, assuming that all activated cells secrete IL-2 with the same rate (Fig. S2A) and 2) Assuming that in addition to the IL2 secretion that depends on TCR activation there is a constant secretion rate that can, for example, be the result of other unrelated cells that create a 'background' IL2 pool (Fig, S2B).

In the first case, the term $s \cdot m$ is in Eq. S1 is replaced with the term, s, while in the second case it is replaced with the term, $s \cdot (m + constant)$ and the initial value of IL2 is $(constant)/d$.

The phase-space diagrams for these two cases are shown in figure S2 and are similar to the result obtained with varying secretion rate (Figs. 3, 4 in the main text). This result implies that the main determinant of interaction is the interplay between the TCR strength and the receptor feedback, and not TCR mediated changes in IL2 secretion levels. This is related the well mixed environment assumption. In this environment, the cells share the same IL-2 pool and are not limited by their own secretion rate. However, cooperation and competition still occurs, due to the dependence of the receptor production rate on m.

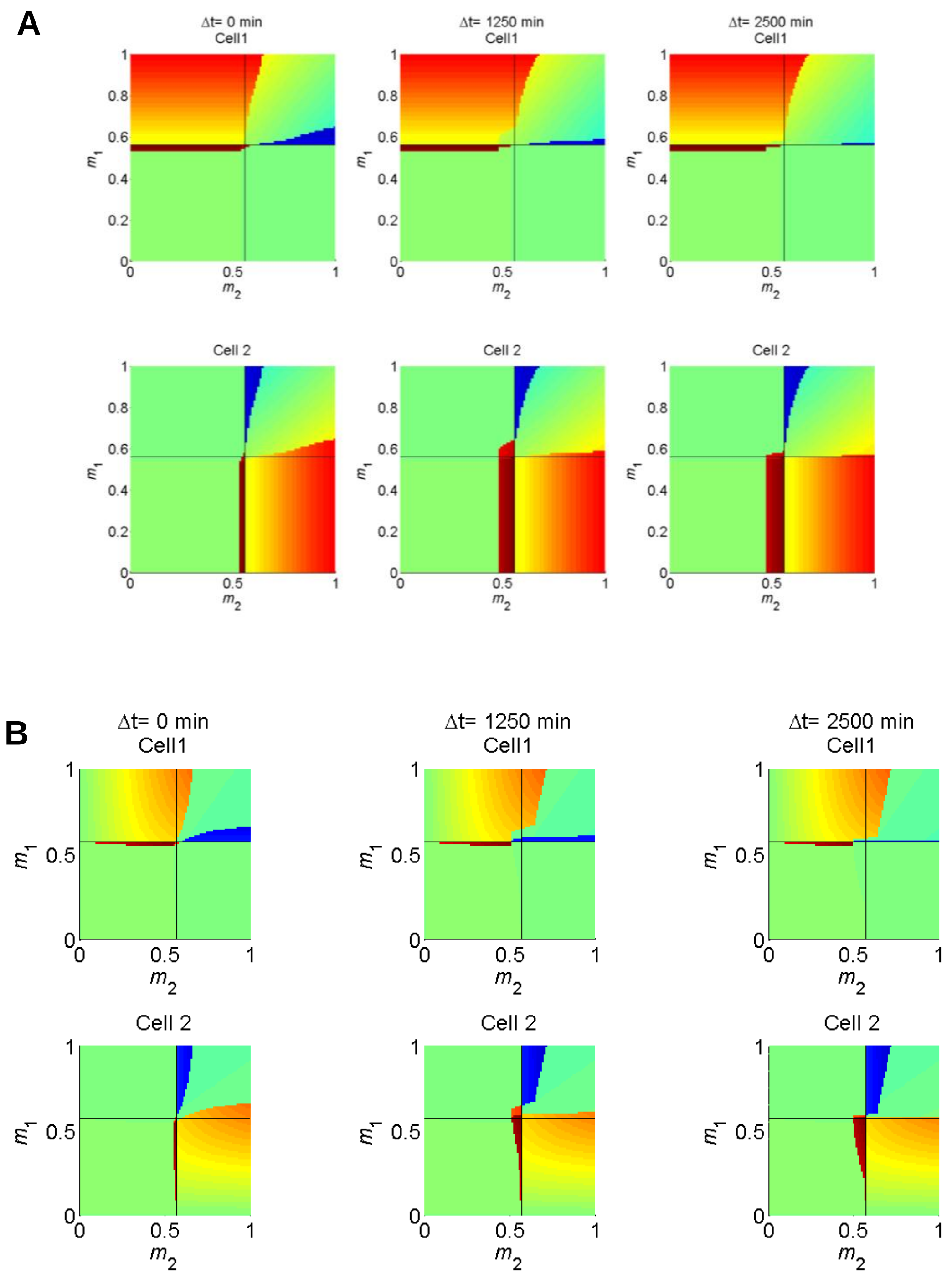

Figure S2: Phase space diagrams for the case of a constant IL-2 secretion rate (A) and the case of adding of a constant secretion rate which is 20% of the maximal secretion rate (B). The normalized interaction index, C (as defined in Eq. 5 in the main text) is presented for both interacting cells (color bar). The black lines are the critical TCR signal strength, m_c, for a single cell. *Cell1* activates Δt minutes before Cell2. As Δt is larger, the area of *Cell2* that can be excluded (blue) increases. In (A) all cells secrete IL-2 with the same rate – the maximal secretion rate, *s* (see table S1); while in (B) the IL-2 secretion rate in Eq. S1, $s \cdot m$, is replaced with $s \cdot m + 0.2 \cdot s$.

5. Sensitivity Analysis

We varied the parameters of the model (table S1). The variation is at least ± 20%. The main effect of changing model parameters is to vary the critical *m* threshold value. However, the basic features of the system, as indicated by the phase space interaction diagrams, are more stable (Figure S3-S9). As the scale for *m* is arbitrarily chosen to be 0-1, the actual range of parameters in which the system shows a similar phase-space diagram, with areas of time-dependent cooperation, co-existence and competition, is larger.

Changing IL-2 secretion and degradation rates have mild effect. As expected, when the level of available IL-2 is higher (higher secretion / lower degradation), the critical threshold value is slightly reduced (Figs. S3,4). In addition, in the case of increased IL-2 secretion rate the exclusion regions for cells above activation threshold are smaller (Fig. S3, top).

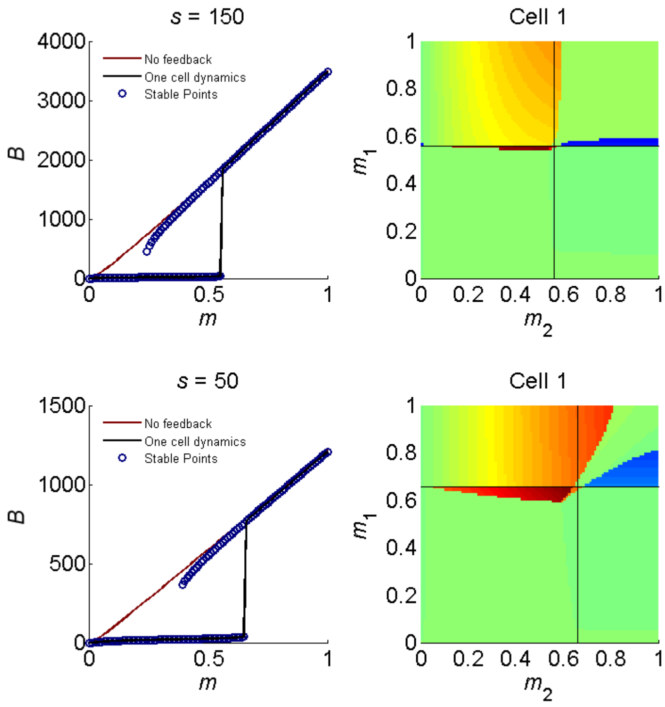

Figure S3: Variation in the IL2 secretion rate, *s*.

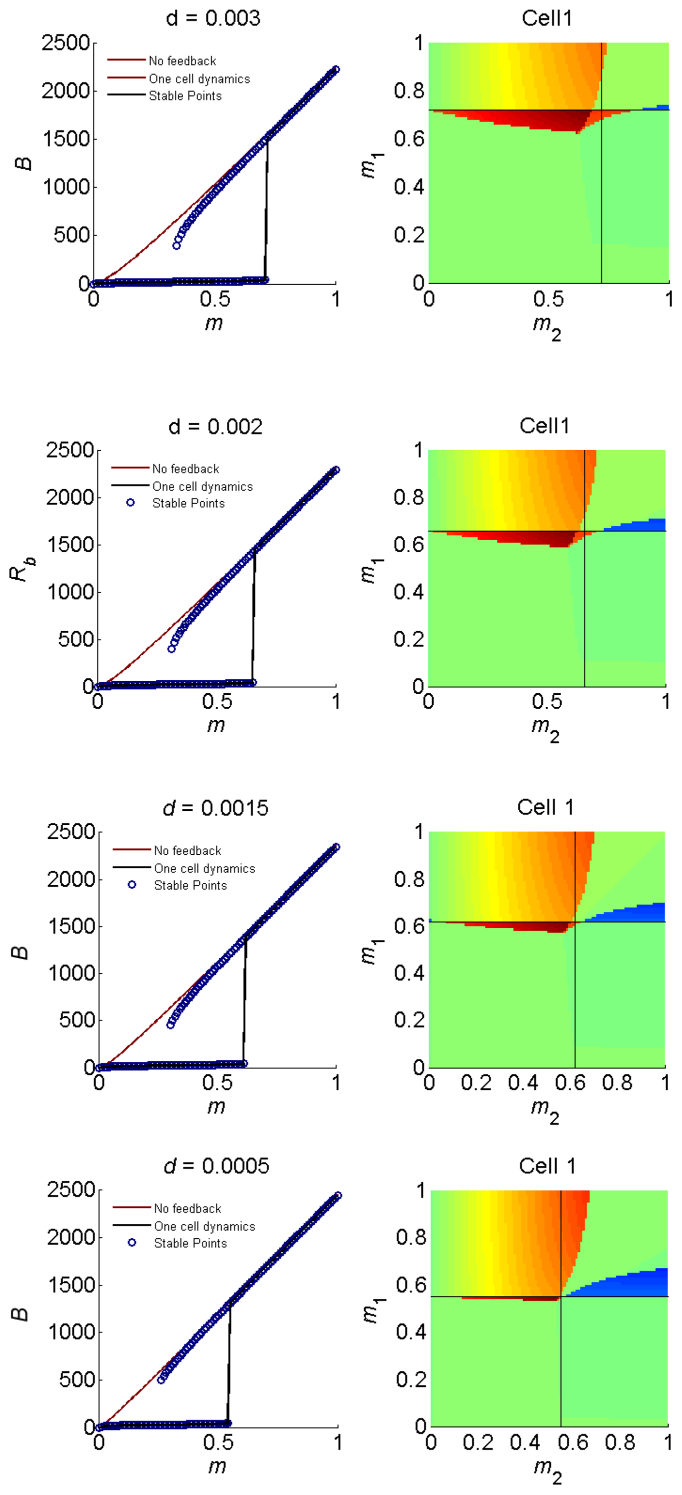

Figure S4: Variation in the IL2 degradation rate, *d*.

We also checked the effect of changing the parameters of constitutive IL-2R dynamics, g_c and a_U while keeping their ratio, which is the initial number of unbound IL-2R, U, fixed (Fig. S5). Reducing / increasing the constitutive synthesis rate results in a higher / lower threshold, respectively. The overall pattern of cooperation / competition remains similar.

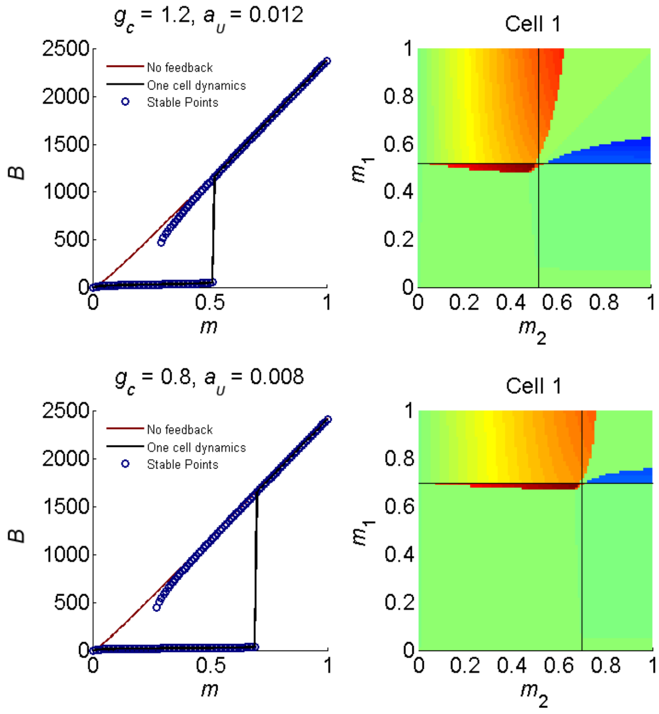

Figure S5: Variation in the constitutive receptor synthesis rate, g_c and in the unbound receptor internalization rate, a_U. The parameters were changed while keeping the number of initial unbound receptors fixed.

The parameters that strongly affect the IL-2R threshold are related to the induced IL-2R synthesis rate, g_f, a_B and K_f (Fig. S6-S8). Reducing the induced synthesis rate, $g_{f,}$ or increasing the induced internalization rate, $a_{B,}$ strongly increases the threshold. Increasing the feedback saturation constant, K_f, has a similar effect to increasing a_B. As above, the overall pattern of cooperation / competition remains similar around the shifted threshold.

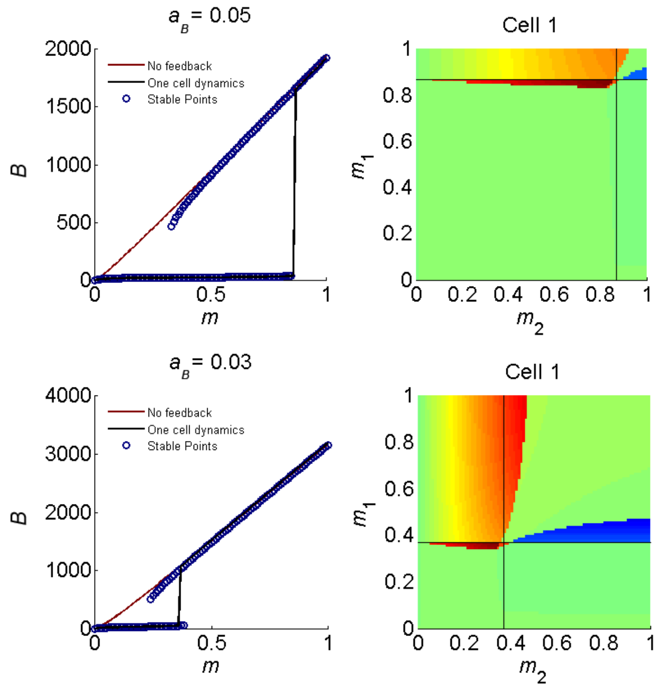

Figure S6: Variation in the bound receptor internalization rate, a_B

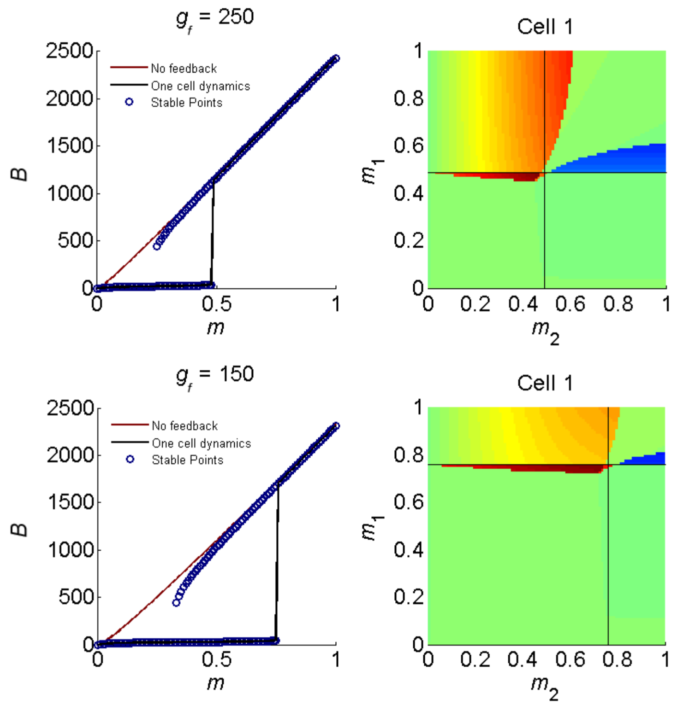

Figure S7: Variation in the maximal induced synthesis rate, g_f.

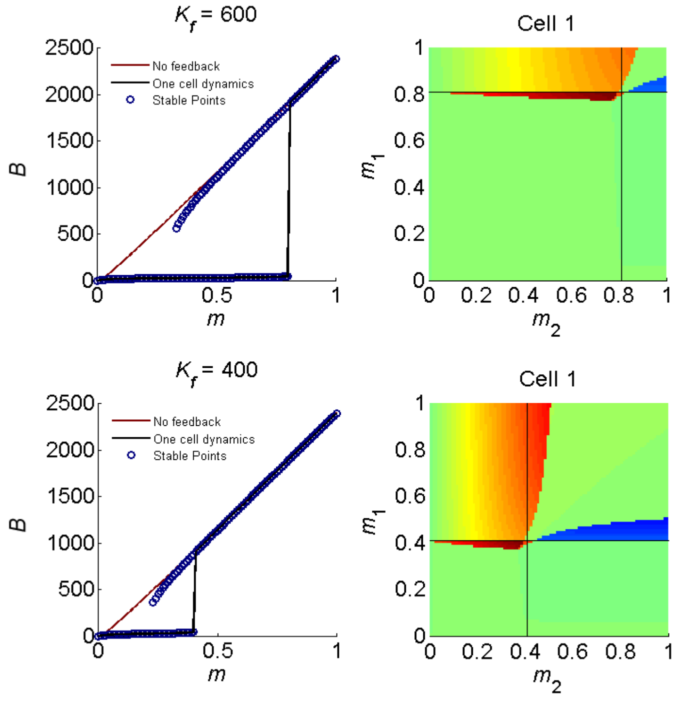

Figure S8: Variation in the feedback threshold level, K_f.

Finally, we check the effect of changing the Hill parameter in the term describing the positive feedback that upregulates the expression of IL-2R. In the main text we used a value oh n = 2. Figure S9 shows that other values of the Hill coefficient, as long as *n* > 1, provide similar behavior of the system of two interacting cells (see also figure 3B in the MS).

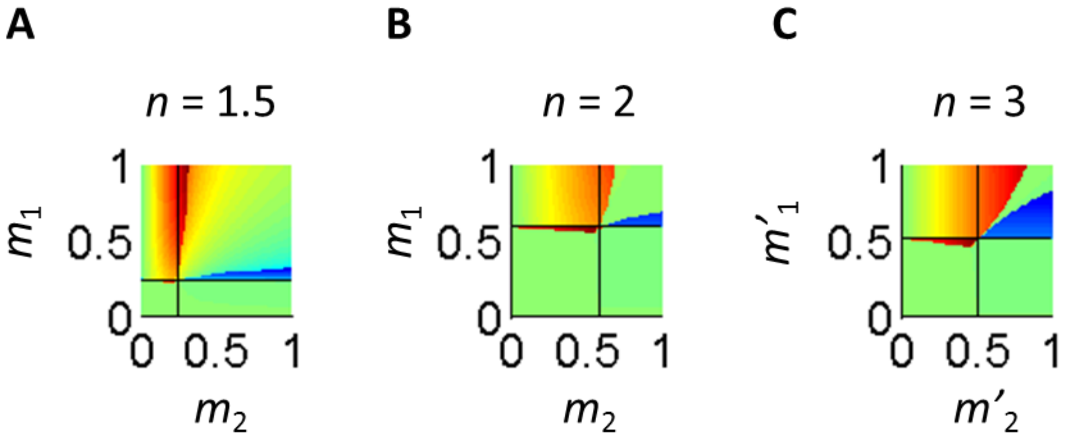

Figure S9: Variation in the Hill coefficient, *n*. The interaction index is shown for *n* = 1.5 (A), *n* = 2 (B) and n = 3 (C). In the case of *n* =3, the value of *m* and the maximal secretion rate, *s*, are scaled (*m'* = m/15, *s'* = *s*/15) to compensate the increase in the critical *m* value.

6. Mapping activation time to initial conditions

An intriguing phenomenon that emerges from our model is the dependence on time of activation. If a strong cell is activated a long time before a mediocre competitor, the weaker cell is excluded. On the other hand, if the mediocre cell is activated a long time before the strong one, they will co-exist. However, in some cases - when the difference between times of activation is small, there could be a scenario in which increasing the time advance of the mediocre cell leads to exclusion (Fig. S10).

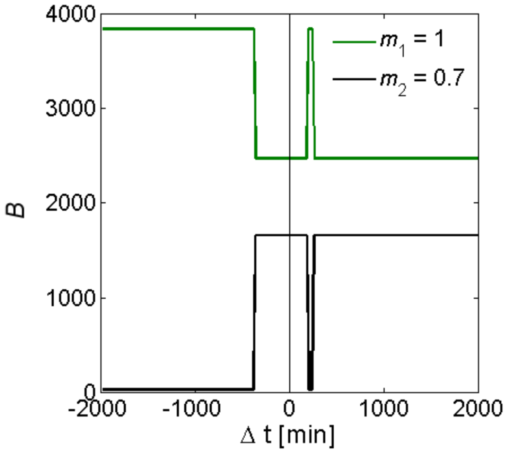

Figure S10: The competition between m_1 = 1 and m_2 = 0.7. Negative Δ*t* represents that the stronger cell is activated first. If the strong cell is activated a long time before the weaker one (Δ*t* << 0), exclusion of the weaker cell occurs. If the weaker cell is activated a long time before the strong one (Δ*t* >>0), there is co-existence. When Δ*t* is around zero, the behavior is more sensitive to initial conditions.

To further understand this behavior, we studied the propagation of cell induction in a three-dimensional phase space that fully describes the dynamics of the system. At each time point, a cell is characterized by its levels of *U* and *B*, and the level of IL-2 in the environment. When the second cell is activated, the fate of the system, exclusion or co-existence, depends on the current values of *I*, *U* and *B*. We plot for the above case (m_1=1, m_2=0.7), the trajectory of a single cell in the 3D concentrations phase-space (black line). The second cell joins at some time, at which the first cell is at a particular point along its trajectory. This point determines the outcome of the interaction. Hence, the phase-space (Fig. S11) is composed of points which results in exclusion (red), and points which results in co-existence (green).

In the case that the strong cell, *m* =1, is activated first (Fig. S11A), the single cell orbit crosses the separator only once. Note that in the region of small concentrations, the orbit is very close to the separator and thus maybe sensitive to changes in initial conditions or to noise. In the case that *m* = 0.7 activates first, the orbit is so close to the separator that it crosses it twice leading to the "spike" which appears in Figure S10. In these cases, where the concentrations are low, that is the difference in time of activation is small, the system is sensitive to changes in initial conditions.

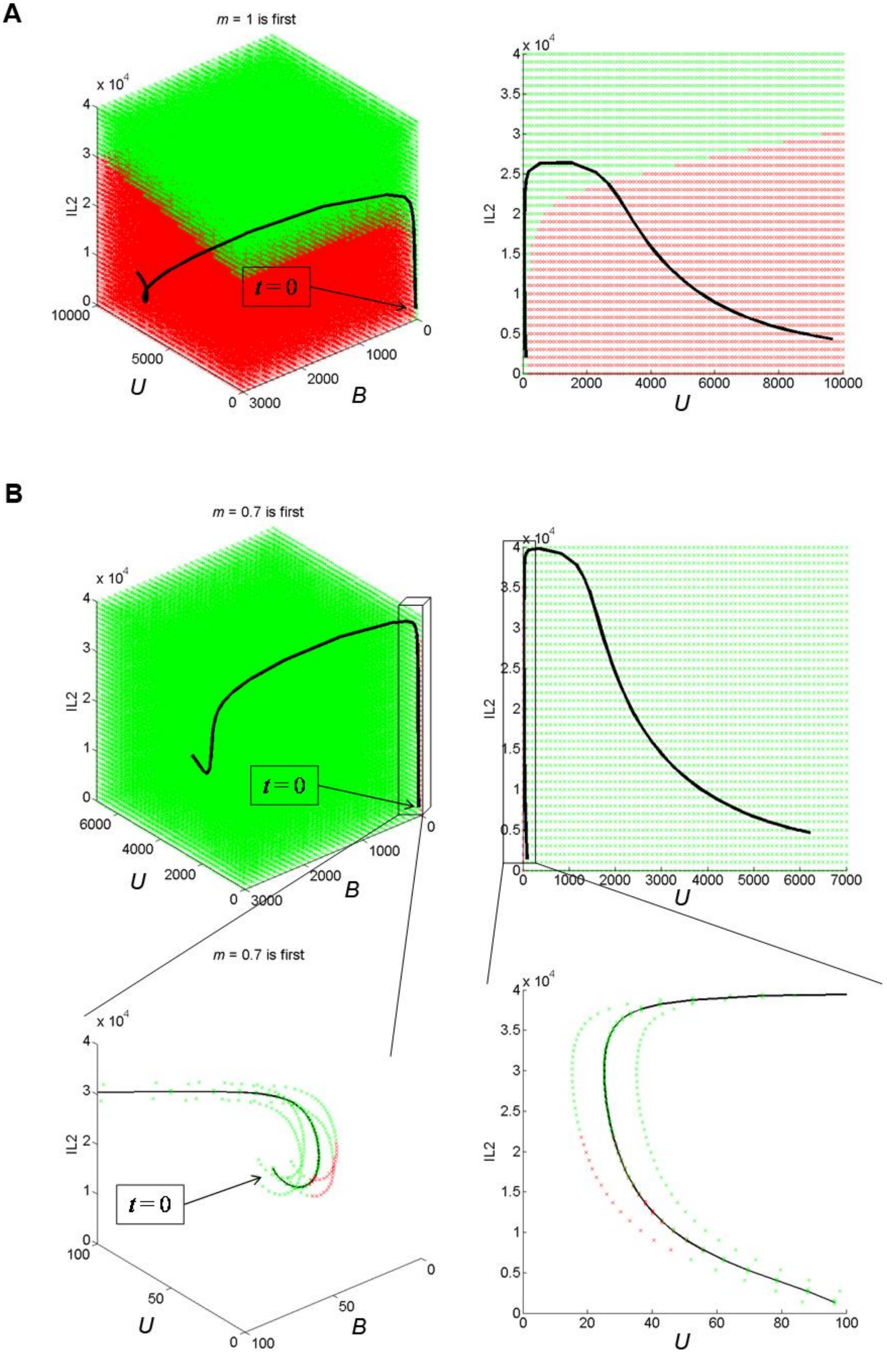
A
m = 1 is first
x 10^4
IL2
U
B
t = 0
B
m = 0.7 is first
x 10^4
IL2
U
B
t = 0
m = 0.7 is first

Figure S11: Dynamical orbits of the competition between *m* = 1 and *m* = 0.7. (A) The stronger cell, *m* = 1, activates first, and its dynamics evolves along the orbit (black line) determined by Eq. (S1). The fate of the system, exclusion of *m* = 0.7 (red) or co-existence (green), depends on the position of *m* = 1 along its orbit at the time of arrival of *m* = 0.7. (B) The weaker cell, *m* = 0.7, activates first. The cross of the phase separator by the orbit is manifested by the 'spike' in figure S10. The lower plate is a "zoom-in" on the separator crossing region. For clarity, we present only a small fraction of the phase space, around the single cell trajectory.

7. The role of IL-2R subunits

In Eq. S1, we consider the dynamics of an *effective* IL-2 receptor. The actual IL-2 receptor is composed out of three subunits, α, β and γ. While activation signal is mediated by a complex of these three subunits, only the α subunit synthesis rate is subject to positive feedback.

The dynamics of the full system, assuming that β and γ vary slowly in time, is given by:

$$\begin{aligned}
&\dot{I} = s \cdot m - d_I I - k_{on,1} \cdot I \cdot \alpha + k_{off,1} \cdot I\alpha \\
&\dot{I\alpha} = k_{on1} \cdot I \cdot \alpha - k_{off1} \cdot I\alpha - k_{on2} \cdot \beta\gamma \cdot I\alpha + k_{off2} \cdot I\alpha\beta\gamma \\
&\dot{I\alpha\beta\gamma} = k_{on2} \cdot \beta\gamma \cdot I\alpha - k_{off2} \cdot I\alpha\beta\gamma - a_1 \cdot I\alpha\beta\gamma \\
&\dot{\alpha} = g_c - a_2 \cdot \alpha - k_{on,1} \cdot I \cdot \alpha + k_{off,1} \cdot I\alpha + m \cdot g_f \frac{(I\alpha\beta\gamma)^2}{(I\alpha\beta\gamma)^2 + K_f^{\,2}}
\end{aligned} \qquad \text{(S5)}$$

The corresponding parameters appear in table S2.

Parameter	Value	Comments
k_{on1}	3.1e^{-6}[min^{-1}]	[2]
k_{off1}	24 [min^{-1}]	[2]
k_{on2}	0.018	[2]
βγ	1000	[2]
k_{off2}	0.0138	[4]
a_1	0.04	[6]

Table S2: Parameters related to the full IL-2R dynamics.

First, we examine the fixed points of this system. In steady state, $I\alpha\beta\gamma = I\alpha \cdot k_{on2}\beta\gamma/(k_{off2} + a_1) = I\alpha \cdot C$ and S5 becomes

$$
\begin{aligned}
0 &= s \cdot m - d_I I - k_{on,1} \cdot I \cdot \alpha + \frac{k_{off,1}}{C} \cdot I\alpha\beta\gamma \\
0 &= k_{on1} \cdot I \cdot \alpha - \frac{k_{off1}}{C} \cdot I\alpha\beta\gamma - a_1 \cdot I\alpha\beta\gamma \\
0 &= g_c - a_2 \cdot \alpha - k_{on,1} \cdot I \cdot \alpha + \frac{k_{off,1}}{C} \cdot I\alpha\beta\gamma + m \cdot g_f \frac{(I\alpha\beta\gamma)^2}{(I\alpha\beta\gamma)^2 + K_f^{\ 2}}
\end{aligned}
\tag{S6}
$$

It is easy to see that these equations are the same as the steady state equations that result from Eq. S1, if we use:

$U = \alpha$; $B = I\alpha\beta\gamma$; $a_1 = a_B$; $a_2 = a_U$; $k_{on} = k_{on,1}$ and $k_{off} = k_{off,1}/C$ (Figs. S12, S13).

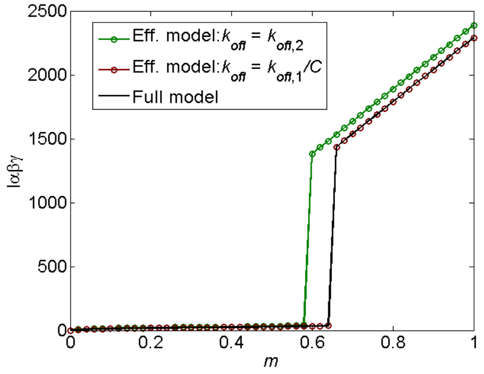

Figure S12: One cell fixed points results for full and effective model. The full model (Eq. S6) and the effective one (Eq. S1) give the same fixed points if $k_{off} = k_{off,1}$/C (see text). In particular, the number of bound signaling receptors as a function of TCR strength *m* of the two models coincide when $k_{off} = k_{off,1}$/C (black and red lines). If $k_{off} = k_{off,2}$, the effective model (green line) yields a slightly different TCR threshold.

Moreover, since $k_{on2}\beta\gamma >> k_{off2}, a_B$, $I\alpha\beta\gamma$ follows rapidly the dynamics of $I\alpha$, $I\alpha\beta\gamma(t) \approx I\alpha(t) \cdot C$. Thus, as long as C >> 1, Eq. S1 describes, to a good approximation, the interaction between two cells as given by the full receptor model (Fig. S12).

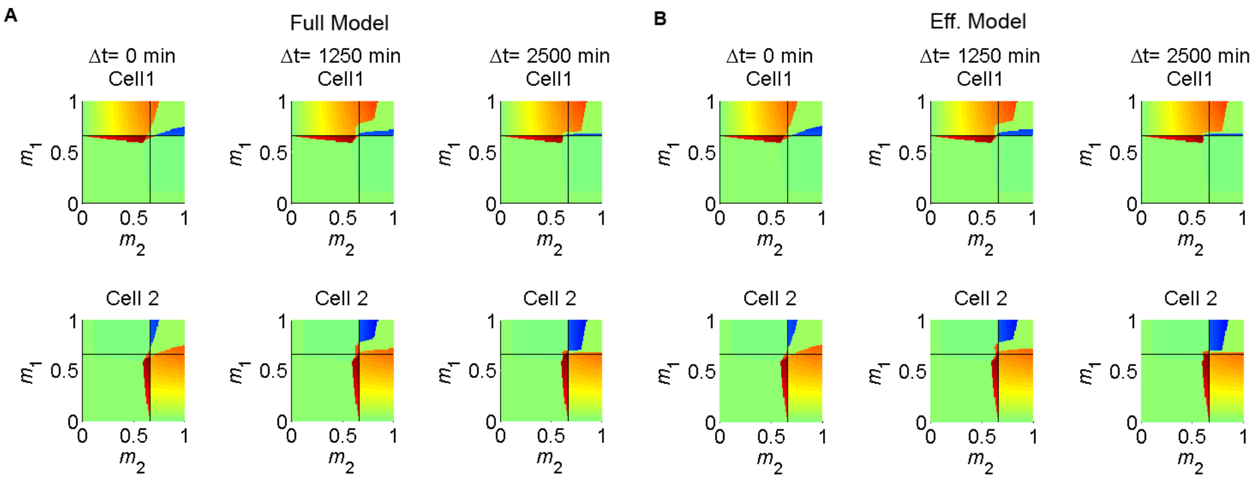

Figure S13: Phase space diagrams of the full and effective models. Phase diagrams for the full model (A), and for the effective model with $k_{off} = k_{off,1}/c$ (B). The resulting interactions between cells are practically identical.

8. Interaction between cells with the same *m* value

When the cells are activated at the same time, interaction between two cells that are slightly below commitment threshold leads to their commitment, akin to quorum sensing (Fig. S14A). When the two cells are activated at different times and the *m* value of both cells is slightly below threshold, the later activated T-cell become committed while using the IL-2 secreted by the earlier activated T-cell which remains uncommitted (Fig. S14B). If both are above threshold, there is a competition between the T-cells that could lead to exclusion of the earlier cell, if the *m* values are slightly above threshold, or the later cell, for intermediate *m* values (Fig. S14B).

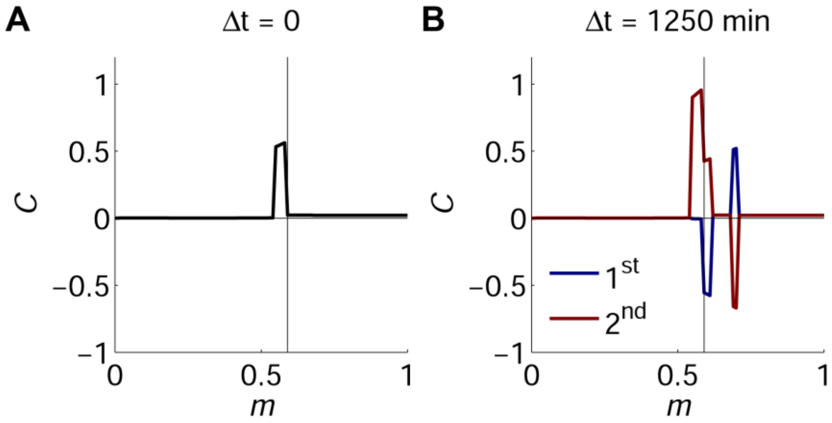

Figure S14: Two cells with the same *m* value exhibit a complex time dependent behavior. Normalized bound IL-2R*, B*, of two cells with the same *m* value. (A) When the two cells are activated at the same time, the interaction results in activation through cooperation in a range of *m* values that are slightly below threshold. The vertical black line marks the activation threshold had the cells been activated alone. (B) When one cell is activated long before the other (Δt = 1250 min), a more complex behavior emerges. If *m* is slightly below threshold, then the 2nd cell (red line) utilizes the IL-2 already secreted by the 1st cell (blue line) and undergoes activation while the 1st cell remains inactivated. If *m* is above threshold, there is a competition between the T-cells that leads to exclusion of the early activated cell if *m* is slightly above threshold or to exclusion of the late activated cell if *m* is at an intermediate range.

9. Interaction between regulatory T-cells (Tregs) and effector T cells

We model Treg dynamics with equations similar to equations S1. In our model we assume two differences between an effector T cell and a Treg: 1) the latter does not secrete IL-2 and 2) has a higher constitutive IL-2R synthesis rate. In our calculation we assumed that the constitutive IL-2R synthesis rate of Tregs synthesis rate is 5 times higher than this of effector cells. We further assume that the non-linear positive feedback is the same in both cell types.

The phase-space map describing the interaction between an effector cell and a regulatory cell is shown in Figure S15. The presence of the Treg strongly reduces the number of bound receptors on the effector cell in a large range of m values, when the two cells are activated at the same time (Fig. S15A). However, this effect diminishes if the Treg is activated after the effector cell. In this case, full inhibition occurs only when the effector cell is not strongly activated (Fig. S15B). It is insightful in this context to examine the result of an interaction between an effector cell and another effector cell that does not secretes IL-2 (Fig S16). As in the case of Tregs, the number bound IL-2R is reduced due to the interaction and this reduction is less significant if the effector cell arrives before its competitor.

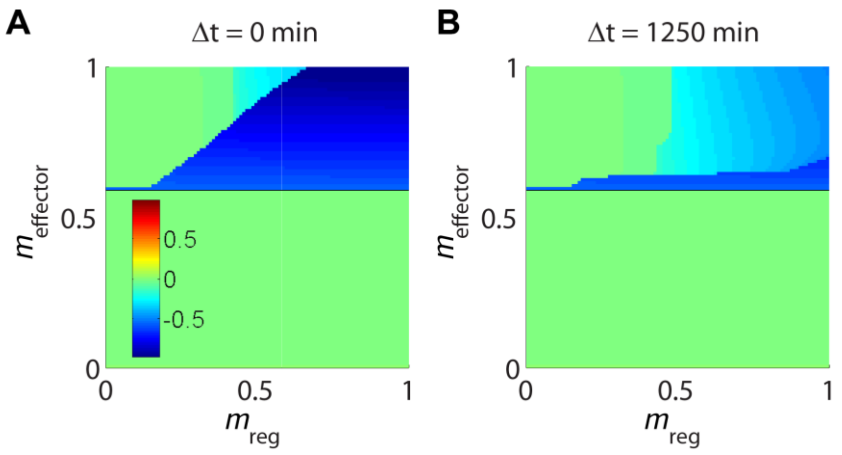

Figure S15: Interaction of an effector T cell with a regulatory T cell is time dependent. Normalized bound IL-2R, *B*, of an effector T cell that is interacting with a Treg cell. (A) When the two cells are activated at the same time, the Treg excludes the T effector cell over a wide range of *m* values since it consumes IL-2 at a high rate due to its high level of IL-2R, but does not secrete IL-2. (B) When the Treg is activated after the T effector cell, inhibition is diminished. Full inhibition is obtained only when the effector T cell is weakly activated with *m* values slightly above threshold. The horizontal black line mark the activation threshold of the T effector cell had it been activated alone. Color bar is the same for both panels.

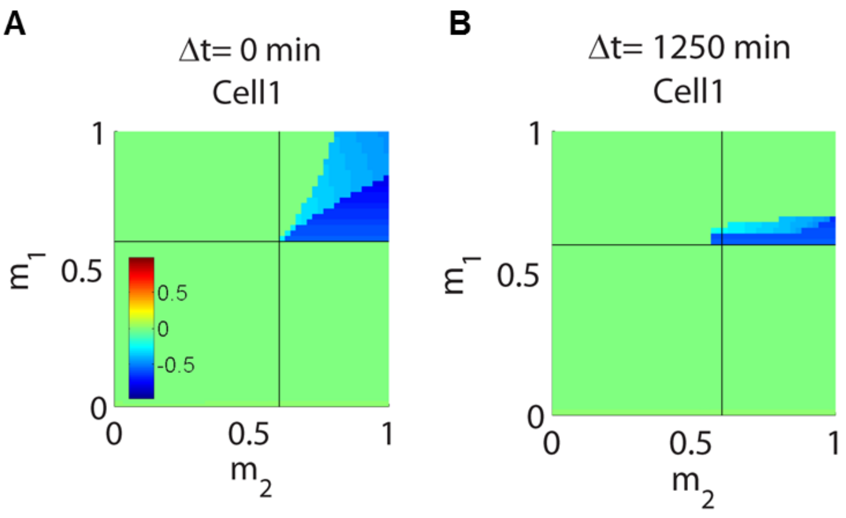

Figure S16: Interaction phase diagrams for an effector T cell that is interacting with a non-secreting T cell. (A) When the two cells are activated at the same time, the non-secreting cell excludes the secreting cell by consuming IL-2 but not contributing any. (B) When the non-secreting cell is activated after the secreting cell, inhibition is reduced.

10. Examples of temporal dynamics

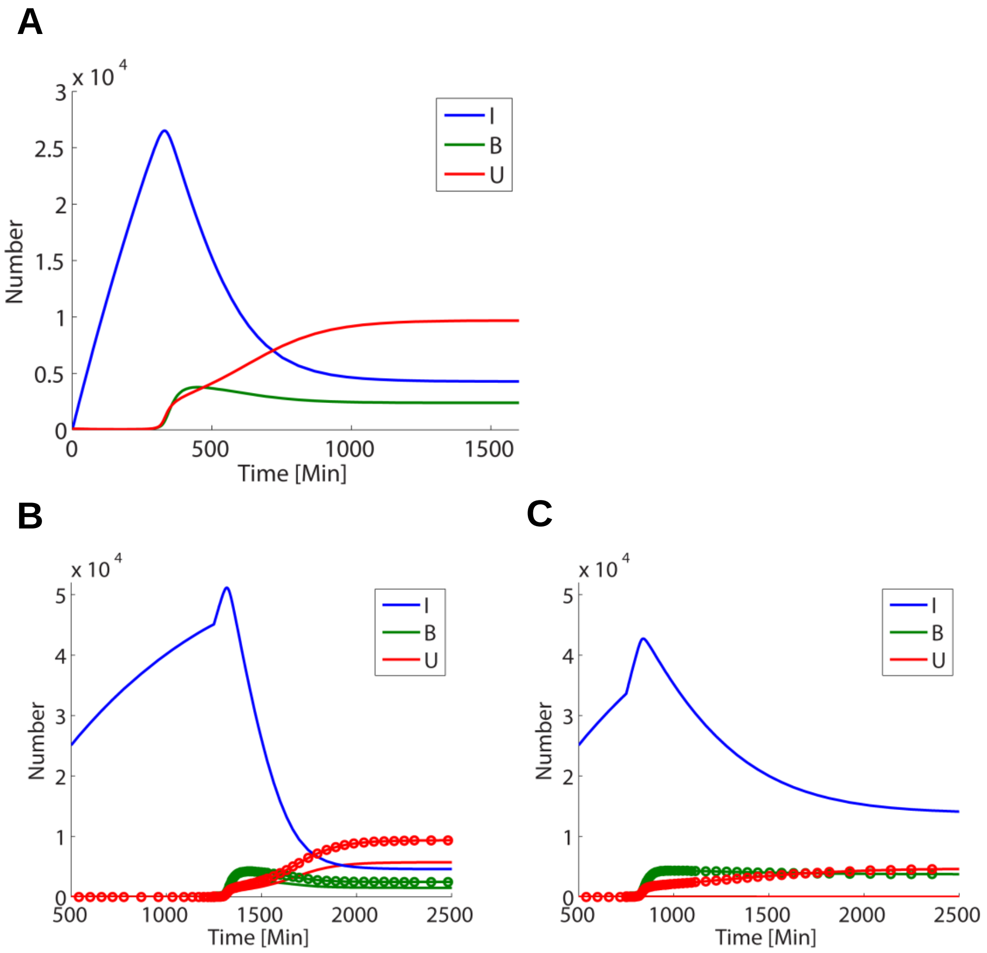

Figure S17: Examples of time dynamics. (A) The time evolution of free IL-2, *I*, bound, *B*, and unbound, *U*, IL-2R for *m* = 1. Zero is the time of TCR activation. (B,C) Interaction between *m* = 1 (circles) and *m* = 0.65 (see also Fig. 4 in the MS). Both cells are above interaction threshold. In both B and C *m* = 1 is activated 1250 and 750 minutes, respectively, after the activation of *m* = 0.65. While in B both cells exhibit IL-2R levels which are similar to levels without interaction, in C the weaker cell IL-2R levels are suppressed.

11. Analytical approximations for activation time and critical *m*

It is insightful to estimate the time it takes the cells to start upregulating their IL-2R. As the cell encounters a cognate antigen it starts secreting IL2. At this stage, the IL-2R levels are low and thus $I(t) = s \cdot m / d\left(1-e^{-d \cdot t}\right)$. The change in IL2 levels is slow relative to the binding, unbinding and internalization and thus we assume that, in this stage, *B* is in quasi-steady state. Equating the 2nd Equation in Eq. S1 to 0, we obtain: $B(t) \approx T(t)\dfrac{I(t)}{I(t)+K}$, where, $K = \left(a_B + k_{off}\right)/k_{on}$, and $T(t)$ is the total number of IL-2R that is around the initial value of g_c / a_U. The positive feedback term, $m \cdot g_f \dfrac{B^2}{B^2 + K_f^2}$, starts to effect when it is comparable to the active degradation term, $a_B B$. At this point, $B << K_f$ and therefore this will happen when $B \approx \dfrac{a_B K_f^2}{m \cdot g_f} = B_0$. For a general hill coefficient *n*, we obtain: $B_0 = \left(\dfrac{a_B K_f^n}{m \cdot g_f}\right)^{1/(n-1)}$. Since at this stage $B(t) \approx g_c / a_U \dfrac{I(t)}{I(t)+K}$, the bound receptor will reach B_0, when $I(t) = \dfrac{B_0 \cdot K}{g_c / a_U - B_0}$.

Using this condition in the equation for *I(t)*, $I(t) = s \cdot m / d\left(1-e^{-d \cdot t}\right)$, solving for *t*, and using the above definition for B_0 (with *n*=2), we find that the activation time is approximated by:

$$t \approx \frac{1}{d}\log[\left(1 - \frac{\dfrac{a_B K_f^2}{m \cdot g_f} \cdot d \cdot K}{s \cdot m(g_c / a_U - \dfrac{a_B K_f^2}{m \cdot g_f})}\right)^{-1}].$$

For m = 1 this gives about 290 minutes and for m = 0.7 about 970 minutes. These estimates agree with the numerical simulations (Fig. S16).

The above derivation also provides an estimation for the critical TCR strength, $m_c \approx \dfrac{a_B K_f^n}{g_f}\left(\dfrac{a_U}{g_c}\right)^{n-1}$. For n = 2, this yields, $m_c \approx \dfrac{a_B K_f^2}{g_f}\left(\dfrac{a_U}{g_c}\right) = 0.5.$

12. Stochasticity in the number of initial IL-2R

Since the number of initial IL-2R is small (around 100, see table S1) and thus prone to fluctuations, in this section we investigate the effect of stochasticity in the initial number of IL-2R on the interaction between the cells. For each iteration in the simulation, the number of initial receptors is drawn out of a normal distribution such that the mean is g_c/a_U = 100 and the standard deviation is 50. If this number is negative it is replaced with zero. Figure S18 shows the result for the three representative points from figure 3 in the MS. In the case of cooperation, points I (Fig. S18A) and II (Fig. S18B), the effect of the fluctuations is negligible. However, in the case of exclusion, point III (Fig. S18C), the result is a bimodal destruction where exclusion happens only in 70% of the interactions.

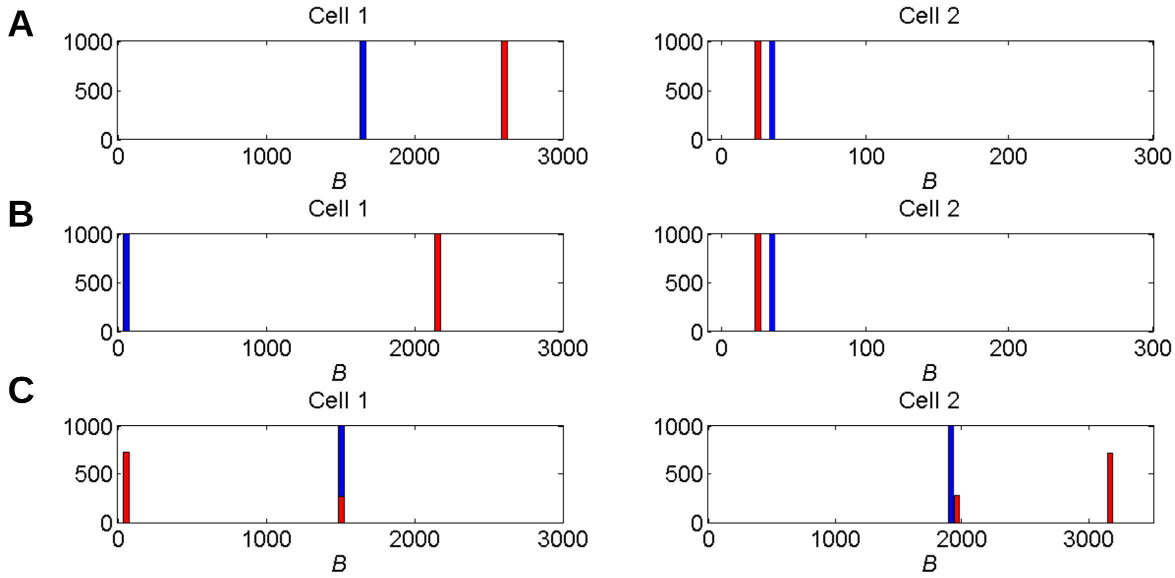

Figure S18: The distribution of *B* when the initial number of IL-2R is noisy. The interaction shown here are the same as the 3 cases shown in figure 3A in the main text, but with random initial levels of U for the two cells. Levels of U were drawn out of a Gaussian distribution with a mean of 100 and SD of 50. Blue denotes the result when the cell is alone and red when the cells are interacting. In the case of cooperation, (A) and (B) the outcome of the interaction is not sensitive to fluctuations in the initial IL-2R. In the case of exclusion (C) a bi-modal distribution emerges and only on about 70% of the interactions exclusion happens.

13. The effect of interaction on the total number of IL-2R

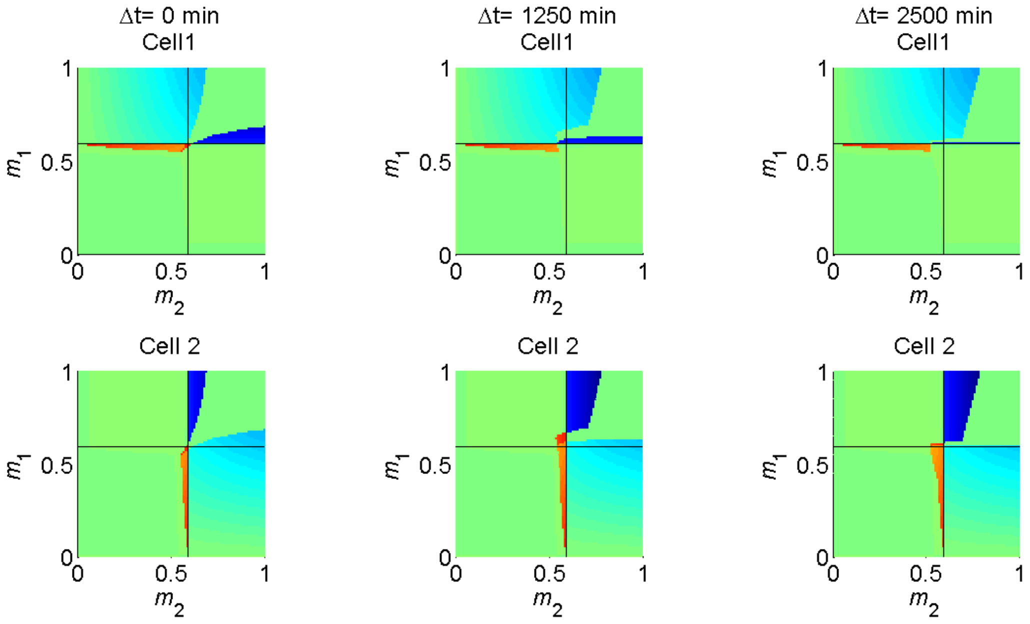

Figure S19: The normalized number of total IL-2R (*B* + *U*), which is defined in a similar way to the interaction index C (as defined in Eq. 5 in the main text). In the case of strong cooperation (point II in Figure 3 in the MS) and exclusion (point III in figure 3 in the MS), the relative change in the total number of IL-2R is similar to the relative change in the number of bound IL-2R. In the case of mild-cooperation (point I in figure 3A in the MS) the number of bound receptor increases while the number of total receptors decreases.

References

1. Sojka, D.K., et al., *IL-2 secretion by CD4+ T cells in vivo is rapid, transient, and influenced by TCR-specific competition.* J Immunol, 2004. **172**(10): p. 6136-43.
2. Feinerman, O., et al., *Single-cell quantification of IL-2 response by effector and regulatory T cells reveals critical plasticity in immune response.* Mol Syst Biol, 2010. **6**: p. 437.
3. Busse, D., et al., *Competing feedback loops shape IL-2 signaling between helper and regulatory T lymphocytes in cellular microenvironments.* Proc Natl Acad Sci U S A, 2010. **107**(7): p. 3058-63.
4. Wang, H.M. and K.A. Smith, *The interleukin 2 receptor. Functional consequences of its bimolecular structure.* J Exp Med, 1987. **166**(4): p. 1055-69.
5. Robb, R.J., A. Munck, and K.A. Smith, *T cell growth factor receptors. Quantitation, specificity, and biological relevance.* J Exp Med, 1981. **154**(5): p. 1455-74.
6. Fallon, E.M., et al., *Increased endosomal sorting of ligand to recycling enhances potency of an interleukin-2 analog.* J Biol Chem, 2000. **275**(10): p. 6790-7.
